# Research trends on parasocial interactions and relationships with media characters. A review of 281 English and German-language studies from 2016 to 2020

**DOI:** 10.3389/fpsyg.2024.1418564

**Published:** 2024-09-26

**Authors:** Holger Schramm, Nicole Liebers, Laurenz Biniak, Franca Dettmar

**Affiliations:** Department of Media and Business Communication, Institute Human-Computer-Media, University of Würzburg, Würzburg, Germany

**Keywords:** parasocial interaction, parasocial relationship, parasocial relationship breakup, review, research trends

## Abstract

Parasocial phenomena are among the most popular and best-researched topics in media reception and effects research. The research can now look back on a history of over 65 years and has experienced another significant boom in recent years. Between 2016 and 2020, more studies were published than in the entire previous 60 years. This descriptive review builds directly on the seminal review by Liebers and Schramm for the years 1956 to 2015 and is based on 281 English-and German-language studies published from 2016 to 2020. The following three research questions guided the review: How are the studies from 2016 to 2020 positioned in terms of the media contexts examined, the parasocial phenomena investigated, the methods and measurements used and the samples? (RQ1) What has changed in the studies from 2016 to 2020 compared to the studies from 1956 to 2015 in terms of the media contexts investigated, the parasocial phenomena studied, the methods and measurements used and the samples? (RQ2) What substantive perspectives and key insights can be gained from the studies conducted between 2016 and 2020 and what gaps in research can still be observed? (RQ3). The results show, for instance, that the largest proportion of studies from 2016 to 2020 focused on the area of social and new media, on non-fictional characters and amicable forms of parasocial relationships, using surveys and existing measurement instruments or adapted versions of them (RQ1). Compared to the studies from 1956 to 2015, parasocial phenomena are increasingly being researched experimentally, as mediating and moderating factors and using established scales, with a slight increase in the proportion of studies specifically investigating parasocial relationships or relationship breakups (RQ2). The research focus has evolved from film and television to social media and cross-media settings. Most empirical studies of parasocial phenomena still rely on young and predominantly female samples (RQ2). The neglect of negatively valenced parasocial phenomena, comparative cultural perspectives, comparative media perspectives, long-term effects and less educated and older people as samples can still be identified as major research gaps (RQ3) and opens up numerous opportunities for future research.

## 1 Introduction

Sixty years after the original essay “Mass communication and para-social interaction: Observations on intimacy at a distance” by Horton and Wohl (1956), over 250 studies of parasocial interactions (PSI) and relationships (PSR) had been conducted by 2015, according to an inventory by Liebers and Schramm (2017a, 2019). Parasocial phenomena have become one of the most popular and well-researched topics within media reception and media effects research, increasingly investigated by several subdisciplines of communication science, including entertainment research, health communication, political communication, and advertising research, but also by disciplines near to communication science, such as marketing, tourism, and human–computer interaction research (Liebers and Schramm, 2023). Social media, through which social and parasocial communications can occur (Hartmann, 2023), have played such a prominent role in the daily lives of most people for years that research into the (para)social phenomena associated with the use and effects of social media—focusing particularly on the “stars” of social media, influencers—has increased markedly in recent years (Liebers and Schramm, 2023). More studies were published between 2016 and 2020 than in the previous 60 years (Liebers and Schramm, 2023). What findings and trends can be derived from these studies? Where are PSI and PSR research heading thematically and methodologically? What topics and research questions remain neglected? To answer these research questions, we have undertaken a descriptive and as complete as possible review of recent research on parasocial phenomena, which builds directly on the fundamental inventory by Liebers and Schramm (2017a, 2019) for the years 1956 to 2015.

## 2 Background and research questions

Research into parasocial phenomena has undergone considerable theoretical and empirical development and differentiation over the last decades. “One of the most important theoretical advances within parasocial research is the differentiation between PSIs (i.e., mediated interactions during media consumption) and PSRs (i.e., overarching relationships that are not limited to media consumption)” (Liebers and Schramm, 2023, p. 17). While PSI and PSR were still used largely undifferentiated in the 1980s – both in theoretical considerations and with regard to empirical measurement approaches – theoretical model-building and methodological work from the 1990s, 2000s and 2010s has led to a fundamental professionalization of the research area (Liebers and Schramm, 2023), so that both parasocial phenomena are even assumed to have different processing and effect mechanisms under certain conditions (e.g., Tukachinsky and Sangalang, 2016).

Despite these further developments, also, for example, with regard to the specific forms of parasocial phenomena (e.g., friendly vs. romantic vs. negative; low vs. high-level; initiation vs. termination), the inventory by Liebers and Schramm (2017a, 2019) showed a sobering picture of the variability of this research area for the first 60 years: more than half of all studies were conducted in the field of “film and television,” but hardly any studies on print media and auditory media. While around 80% of the studies examined parasocial phenomena in the classic way, either as a starting point/independent variable or as an endpoint/dependent variable, not even 10% of the studies attempted to see parasocial phenomena as playing the mediating or moderating role that they are likely to play in most media usage contexts. Almost two thirds of the studies used a survey method (without an experimental design), and not even one in five studies was an experiment. In other words, most of the studies had a correlative study design, which cannot empirically trace clear cause-and-effect mechanisms. In addition, within the studies that quantified PSI and PSR, the majority used an established scale or an adaptation of such a scale – and in most cases this was the short or long version of the PSI scale (Rubin et al., 1985; Rubin and Perse, 1987), which was used in 95 studies. However, this scale does not sufficiently differentiate between PSI and PSR (Schramm and Hartmann, 2008; Dibble et al., 2023), meaning that the parasocial phenomena were not measured precisely and validly enough in a large number of the studies from the first 60 years, which was derived as a major learning from this first inventory. What also turned out to be deficient were the samples that were frequently used in the studies of the first 60 years: they were predominantly student, female and young samples, whereas older and uneducated people were hardly studied at all (Liebers and Schramm, 2017a, 2019).

This detailed information has shown what and how research was carried out in this field of research in the first 60 years and what was not taken into account. We see it as a sensible task to continue this work in order to be able to show whether—and if so, how—this research has developed further and become more differentiated. To this end, we first want to take a look at the research of the following five years, especially as this research alone is already more extensive than that of the previous 60 years. In a first step, we want to systematically record and describe the status quo of this research in the same way as in the review by Liebers and Schramm (2017a, 2019). In a second step, the results of this analysis will be compared with the results of the analysis of the first inventory in order to gain insights into what has changed in these five years compared to the research of the previous 60 years. In a third and final step, we want to delve a little deeper into the research of these five years and ask what perspectives and key insights can be gained from the studies and what is still not being investigated and therefore remains a research desideratum. Consequently, the following three research questions guide our work.

RQ1: How are the studies from 2016 to 2020 positioned in terms of the media contexts examined, the parasocial phenomena investigated, the methods and measurements used and the samples?

RQ2: What has changed in the studies from 2016 to 2020 compared to the studies from 1956 to 2015 in terms of the media contexts investigated, the parasocial phenomena studied, the methods and measurements used and the samples?

RQ3: What substantive perspectives and key insights can be gained from the studies conducted between 2016 and 2020 and what gaps in research can still be observed?

## 3 Procedure/method

The following section describes the methodological approach from the identification to the selection and exclusion of certain publications as well as the systematization and analysis of the included studies from the years 2016 to 2020. In order to maintain a high level of comparability, the methodological approach is based on that of Liebers and Schramm (2017a, 2019).

### 3.1 Identification of publications

Since the beginning of research into parasocial phenomena, most of the work published on this topic has been in English. However, following Liebers and Schramm (2017a, 2019), German-language sources were also included, as these have often attracted a great deal of attention in the international research community in the past (e.g., Gleich, 1997; Hartmann et al., 2004; Vorderer, 1996) and because, within those communities that have repeatedly initiated research on parasocial phenomena for decades, the German community is, to our knowledge, the only one that still writes some of this research in its national language (also because there are still German-language communication science journals in Germany due to the size of the market). Accordingly, both English-language and German-language sources were researched. In order to find as many works published on the topic of “parasocial phenomena” as possible, research was conducted using various English and German terms. This was necessary because the naming or spelling of parasocial phenomena varies across different publications [see, for example, “para-social” in Horton and Wohl (1956) versus “parasocial” in Rubin et al. (1985) versus “para-friendship” in Tukachinsky (2010)]. The following terms were ultimately used in the research: “parasocial,” “para-social,” “parasozial,” “para-sozial,” “para-love,” “para-friendship” and “para-romantic”.

The actual research using the search terms was carried out in the databases *PsycINFO*, *Science Direct* and *Google Scholar*. Each term was entered individually into the search mask, and the databases were searched separately for each of the five years – starting in 2016 up to and including 2020. Each hit was assessed and a decision was then made on the basis of certain eligibility criteria, which are described in the following subchapter, as to whether or not the publication should be included in the subsequent analyses (first ten pages of the displayed results in *Google Scholar* were assessed). A first search took place in December 2020. From April to June 2021, a second wave of searches was carried out for comparison purposes, in which a person not involved in the first wave again checked whether all relevant sources had been included.

### 3.2 Selection and exclusion of publications

In order to ensure that the studies were selected as objectively as possible, several formal and content-related selection and exclusion criteria for the publications were formulated at the start of the research. These followed the criteria of Liebers and Schramm (2017a, 2019) and are explained in more detail below.

In order to ensure a certain standard and also to avoid duplication, only studies that were published in the traditional sense were formally included. This criterion includes journal articles or contributions to anthologies. In addition, monographs such as published dissertations were also included. Unpublished Bachelor’s and Master’s theses and dissertations, on the other hand, were excluded from the outset. Conference presentations and contributions in proceedings volumes were also excluded, as the inclusion of these was feared to result in a high duplication of studies that were first presented at a conference and subsequently published as journal articles, for example. In addition, the respective publication had to have been published for the first time in the years 2016 to 2020. This also means that studies are included in the final analyses that were published in 2021 or even 2022 according to their current literature citation if they were published “Online First” for the first time in 2020 (cf. e.g. Waggoner, 2022).

In terms of content, a key inclusion criterion was that the publication must contain its own empirical study. Purely theoretical contributions (see, e.g., Erickson et al., 2018) or meta-analyses (see, e.g., Tukachinsky et al., 2020) were excluded accordingly and not included in the subsequent analyses. An additional requirement for the inclusion of a publication in the further analyses was that one of the search terms from the search must be found in the title of the publication, the keywords, the abstract or—if not available—the introduction. This was to ensure that a parasocial phenomenon was a central component of the study. The last content criterion relates to the understanding of parasocial phenomena in the study. Following Horton and Wohl (1956), parasocial phenomena describe media-mediated interactions or relationships between real people and media characters. Following this basic assumption, only publications that have examined parasocial phenomena in the context of recipients and media characters in the sense of social entities have been included in this overview. Publications that were consistent with the other criteria in terms of form and content, but which, for example, examined the “parasocial” bond between a media user and a brand, were excluded according to this criterion because they do not understand PSI as an interaction with a media character (cf. e.g. Kim and Kim, 2018).

If a publication met all of these criteria, it was included in the pool of studies. A total of 281 studies (see Appendix 1) were included and further analyzed – including nine book chapters (5 English-language, 4 German-language) and two German-language journal articles (but no monograph).

### 3.3 Systematic analysis of the studies

The 281 studies were coded according to a fixed scheme (see Appendix 2). The dimensions and categories were again taken from Liebers and Schramm (2017a, 2019) and only updated or supplemented in a few details. These included the media context being investigated (film and television; print media; radio, music, and podcasts; social and new media; media cross-cutting contexts), the type of media characters investigated (e.g., fictional vs. nonfictional vs. fictional and nonfictional), the type of parasocial construct studied (PSI, PSR, parasocial relationship breakup [PSRB]), the position of the investigated parasocial construct in the hypothesis model (independent variable, dependent variable, mediator/moderator), the method used (survey, content analysis, experiment, qualitative methods), the type of measurements made of the parasocial construct studied (e.g., the use of an established scale vs. self-formulated items), the extent of the measurements (number of items), and the composition of the sample (by age and gender). Finally, the central research questions and results were recorded for each study, so that an overview of the perspectives and results of the studies from these five years is possible.

Of course, particular attention was paid to the exact coding of the parasocial phenomenon, which is why a distinction was made not only between PSI and PSR, but also between friendly, negative and romantic forms. If several different parasocial phenomena were investigated in one publication, this was also coded. In order to achieve the most accurate coding possible, it could also happen that we did not always code the construct that the respective authors specified for the study. For example, if a study claimed to have investigated PSI, but it was clear from the study context and the type of measurement that PSR was investigated, then we coded “PSR” and not “PSI”.

The two coders were experienced Master’s students and were trained several times until they each coded five studies accurately and identically. Studies that were particularly difficult to code were marked by the coders and then recoded by the two experienced study leaders.

The references of all studies, the coding book for the analysis and the final data can be found on this page of the Open Science Framework (OSF): https://osf.io/7gac4/.

## 4 Results

### 4.1 Setting of the studies from 2016 to 2020 in terms of the media contexts studied, the parasocial phenomena investigated, the methods and measurements used and the samples (RQ1)

#### 4.1.1 Media contexts and parasocial phenomena

The largest proportion of studies (40%) focused on the area of social and new media. Cross-media study contexts occurred in at least one in four studies (27%). This high proportion is also partly due to the high importance of social media in these contexts, as many media users first encounter their favorite characters in media contexts outside of social media, such as podcasts, music streaming services or film and television, and then follow them on social media. The context of film and television is only found in a quarter of the studies (25%), while studies in the context of print media (5%) and auditory media – radio, music and podcasts – (3%) were very rare.

The proportion of studies on parasocial phenomena involving non-fictional media characters compared to fictional media characters is skewed in favor of non-fictional characters due to the large number of studies conducted on social media: 65% of the 281 studies examined parasocial phenomena involving non-fictional media characters, and only 22% of the studies examined those involving fictional characters. The remaining 13% investigated parasocial phenomena involving both non-fictional and fictional characters.

PSR was investigated in 69.8% of the 281 studies, whereas PSI was studied in 34.9% of the studies (cumulative total > 100%, indicating that in some studies, both PSR and PSI were measured). In 7.1% of the studies, the focus was on the consequences and implications of the termination of PSR (PSRB).

97.5% of the studies still almost exclusively examined amicable forms of PSI and PSR, and only 1.8% (five studies) examined negative forms. 3.9% (11 studies) examined romantic PSI or PSR, thus addressing a particular manifestation or play of positively valenced parasocial phenomena that has been popular since Tukachinsky’s (2010) publication 12 years ago.

In nearly one-third of the studies (32%), parasocial phenomena were considered as mediating or moderating variables in the hypothesis model, 22% of the studies investigated parasocial phenomena as a cause, and 20% as an effect.

#### 4.1.2 Methods, measurements and samples

The majority (85.4%) of the 281 studies were based on a purely quantitative approach. Moreover, 11.4% of the studies used qualitative methods, whereas only a fraction (3.2%) used both quantitative and qualitative research methods.

In terms of the data collection methods, there was a strong preference for surveys (59.1%). The second most frequently used method was the experiment (24.6%). Content analysis was a third major method, used to investigate parasocial phenomena in 10.3% of studies.

Most studies (53.7%) relied on a single existing measurement instrument or an adapted version of it, whereas in 18.1% of studies, multiple or various instruments were used. In 2.1% of studies, a few original items were added to an existing measurement instrument. In total, 73.9% of studies relied largely on measurement instruments from previous studies. The PSI Scale (Rubin et al., 1985; Rubin and Perse, 1987) continues to be the most frequently used, appearing in 86 studies, followed by the PSI Process Scales (Schramm and Hartmann, 2008), used in 21 studies, the Audience Persona Interaction Scale (Auter and Palmgreen, 2000), used in 18 studies, the Celebrity Persona Parasocial Interaction Scale (Bocarnea and Brown, 2007), used in 16 studies, the Experience of Parasocial Interaction Scale (Hartmann and Goldhoorn, 2011), used in 13 studies, and the Multiple Parasocial Relationships Scale (Tukachinsky, 2010), used in 12 studies.

In 252 of the 281 studies, individuals were examined and the corresponding personal characteristics collected. In relation to the average age of the majority of participants in these studies, studies of prospective adults, aged 18–24, were most frequent (31.7%), followed by studies of young adults, aged 25–34 (15.5%). In contrast, few studies tested their assumptions on infants or children (2.8%), adolescents (3.2%), or older adults (1.2%). 26.2% of the studies included adults without age information as a sample. It is to be feared that these samples also consisted mainly of young adults, who are the easiest to recruit for scientific studies, especially if the survey takes place online. A clear tendency was observed in the composition of the samples according to the gender of the participants. Only 23% of the samples had a balanced ratio of male and female participants, whereas the proportion of females predominated in 42.8% of the studies. In comparison, only 7.9% of the studies showed a male predominance. Furthermore, 10.7% of the studies investigated samples consisting exclusively of female participants. In comparison, only two studies (0.8%) consisted exclusively of male samples. Fortunately, the gender ratio was unclear in only a very few studies (5.6%), or hypothesis testing was conducted on multiple samples with different gender compositions (7.5%). Therefore, most empirical studies of parasocial phenomena still rely on young and predominantly female samples, and extrapolation of the findings to the general population is not always possible because of the limited representativeness of the samples.

### 4.2 Changes in the studies from 2016 to 2020 compared to the studies from 1956 to 2015 in terms of the media contexts studied, the parasocial phenomena investigated, the methods and measurements used and the samples (RQ2)

#### 4.2.1 Media contexts and parasocial phenomena

While 50.6% of studies from 1956–2015 still examined film and television, this proportion has now fallen sharply to 25%. In contrast, the proportion of studies in the area of social and new media has increased from 17.2 to 40%, and the proportion of cross-media study settings has also risen slightly from 24.8 to 27%. Print media and auditory media remain almost unchanged in their low share, meaning that what film and television have lost in share in the studies in these five years compared to the previous 60 years, social and new media have gained. However, PSI and PSR research still has its home base in audiovisual media, as social media also function via both auditory and visual aspects of use.

In terms of the type of media character being researched, research has shifted further towards non-fictional media characters in line with the shift in focus towards social media. While between 1956–2015, 47.3% of studies researched non-fictional characters, between 2016 and 2020 this figure has risen to 65%, while the proportion of studies on fictional characters fell from 25.2 to 22% and the proportion of studies that researched both non-fictional and fictional characters even fell from 27.5 to 13%.

With regard to the construct examined, the scales seem to tilt somewhat more in the direction of PSR research: In the first 60 years, PSR was the focus of just over half of the studies (53.1%) and PSI in less than a third of the studies (30.5%). Looking at the studies from the years 2016–2020, the proportion has now increased significantly to 69.8% in the case of PSR, although it has also risen slightly to 34.9% in the case of PSI. At second glance, it must be admitted that the figures are only comparable to a limited extent because—as already explained above—double coding per study was possible in this dimension in the years 2016–2020 in order to avoid the category “Multiple constructs” from the analysis of the years 1956–2015. It is particularly interesting that the proportion of studies investigating parasocial breakups has risen massively from 1.5 to 7.1%, which indicates that fragile parasocial phenomena are increasingly coming to the fore.

The studies continued to examine almost exclusively “positive” forms of PSI and PSR (friendship, romance), but in comparison to the studies from the first 60 years, these positive forms were now also examined with regard to antiheroes, villains and questionable media characters (e.g., Oliver et al., 2019). Compared to the first 60 years, five studies even examined negative forms of PSI and PSR, which is almost “nothing” compared to the large number of all studies and therefore represents one of the major desiderata of the research area.

The proportion of studies that have investigated parasocial phenomena in a mediating or moderating function has almost tripled (9.5%= > 32%); consequently, the proportion of studies that have investigated PSI and PSR in the classic way as a cause/independent variable (37.8%= > 22%) or as an effect/dependent variable (42%= > 20%) has fallen massively. The trend is therefore moving towards more complex hypothesis models, which in most cases probably also correspond to the “reality” of processing and which an increasing number of researchers no longer seem to shy away from due to the simplified possibilities of analysis in the last ten years.

#### 4.2.2 Methods, measurements, and samples

The proportion of studies using a purely quantitative approach rose from 81.3 to 85.4%, while the proportion using a purely qualitative approach fell from 13.7 to 11.4%, and the proportion using both quantitative and qualitative research methods also lost ground again, falling from 5.0 to 3.2%. Clearly, research on parasocial phenomena is increasingly dominated by quantitative research.

With regard to the methods used, the survey has lost some of its significance at a high level (64.1%= > 59.1%), whereas the experiment is being used more and more frequently (16.8%= > 24.6%). The importance of content analysis has decreased slightly (12.6%= > 10.3%). This fits in with the above finding that parasocial phenomena are increasingly seen as having a mediating and moderating function, which is usually investigated in concrete reception situations—and these are mostly manipulated experimentally in order to demonstrate clear cause-and-effect principles.

Both in the first 60 years and in the years 2016–2020, around three quarters of the studies largely relied on measurements from previous studies and thus on established measurements—so nothing has changed here at a high level. What has changed, however, are the measurement instruments used. The PSI Scale (Rubin et al., 1985; Rubin and Perse, 1987) is still used very frequently, but its use is declining (36.4%= > 30.6%), while all other frequently used scales (cf. Section 4.1.2) have gained in importance, e.g., the PSI Process Scales (Schramm and Hartmann, 2008; 3.1%= > 7.5%). On the one hand, this is of course also due to the fact that these scales are younger than the PSI Scale and therefore did not even exist in the majority of the years 1956–2015, but it is certainly also due to the fact that researchers are becoming increasingly precise in their construct definition and thus in their measurement.

The sample compositions have only changed slightly for the better and are still too young and too female (and, as they are mostly recruited at universities, also too educated). Studies with a predominance of female participants have declined slightly (43.6%= > 42.8%), while studies with a balanced ratio of male and female participants are now more common (17.6%= > 23%). Fortunately, the proportion of studies in which the gender ratio was not clearly visible has decreased significantly (20.3%= > 5.6%), which indicates greater transparency and thus professionalization of the research field.

### 4.3 Perspectives, key insights, and research gaps of the studies from 2016 to 2020 (RQ3)

Although Liebers and Schramm (2017a, 2019) do not differentiate the perspectives, findings, and research gaps by media, but draw a cross-media overall picture for this period, we consider a media-specific presentation to be useful at this point. For instance, in print media, as well as in purely auditory media, media characters have limited opportunities to address the audience. Therefore, other factors may play a central role in the development of PSI and PSR in these contexts. Furthermore, within the category of audiovisual media, a distinction should be made between film and television on the one hand, and social and new media on the other, because differences in the interactivity factor and the perceived closeness between personae and media users could result in vastly different findings for social media and traditional television.

#### 4.3.1 Parasocial phenomena in the use of print media

The 14 studies conducted between 2016 and 2020 that can be situated in the domain of print media display such diversity in their disciplinary perspectives, methodological approaches, research questions, and media contexts that an overarching summary of their findings would inevitably consist of a concatenation of individual findings. Therefore, only a few conspicuous features and peculiarities are noted.

When parasocial phenomena are considered in the context of print media, PSI and PSR are typically associated with fictional media characters from books or novels. However, within the 14 studies in this field, only three studies addressed this topic (Brodie and Ingram, 2021; Kim and Harwood, 2019; Liebers and Schramm, 2017b). For instance, Brodie and Ingram (2021) surveyed young adults about their PSR with heroes or villains in their favorite comic books and attempted to explain the strength of the PSR through the individual characteristics of the Dark Triad of personality traits (narcissism, psychopathy, and Machiavellianism). However, it is noteworthy that the research conducted in this study did not specifically examine aspects unique to print media or book reading. The research questions could be answered equally well by exploring the favored hero/heroine or villain in an animated television series. This is quite different in the study by Liebers and Schramm (2017b). They investigated the extent to which the characteristics of a book character and the reading experience foster the development of friendly and romantic parasocial relationships. The focus here is on the reading experience or reading pleasure (as it was operationalized), which can only come into play in the reception of print media and which can therefore contribute to very specific explanations of parasocial phenomena in reading, which cannot simply be transferred in this form to other media contexts.

Of the 14 recent studies conducted on the print media context, the remaining 11 did not examine anything inherently unique to print media. The use of (fictitious) newspaper articles or print advertisements as stimuli is justified by their easy use in experimental situations and their manipulability. However, investigating specific parasocial phenomena in the context of print media does not appear to have been the primary research interest. The finding that a fictional character, such as Heidi, used in a print advertisement promoting Switzerland as a travel destination can positively affect the intention of the recipient to visit Switzerland through a parasocial bond (Hosany et al., 2020) is valuable. However, similar results could probably be obtained in other advertising media contexts. Instead, it would be of greater interest to investigate the types of associations with Heidi or the memories of the character that elicit specific effects, or to determine whether the nature and intensity of these associations or memories vary across different media contexts using additional design options, such as by incorporating the well-known melody from the TV series “Heidi” as a memory anchor through an auditory channel.

#### 4.3.2 Parasocial phenomena in the use of radio, music, and podcasts

Eight studies conducted between 2016 and 2020 in the context of purely auditory media showed a high degree of congruence, allowing a cohesive, comprehensive picture to be drawn, because many of these studies emphasized factors that are particularly relevant to auditory media, such as the human voice.

According to the study by Vinney and Vinney (2017), voice recognition in audio formats is a crucial factor in the positive evaluation of those voices, which we also know from other contexts. What the listener knows or recognizes is often evaluated positively or leaves a positive feeling simply because it is familiar and can be processed more fluently (the “fluency effect,” e.g., Reber et al., 1998). According to the results of the study by Vinney and Vinney (2017), the positive evaluation of characters’ voices in audio formats can increase the PSI with the characters and the feeling of transportation among listeners. In addition to voice, it is crucial for the strength of PSI with radio presenters that they appear sympathetic and competent to listeners, according to the results of Spangardt et al. (2016). If this is the case, not only are intense parasocial processes triggered while listening to the radio, but listeners also develop a bond with the presenter, so that they specifically tune in to the shows of those presenters and also follow them on social media. The latter has been confirmed by the findings of Quintero Johnson and Patnoe-Woodley (2016), who showed that the positive perception, memory, and purchase of brands, products, and services recommended by listeners’ favorite radio personalities can be influenced by more-intense PSI and PSR. A study by Gregg (2018) demonstrated that the dismissal of a beloved radio personality can lead to painful PSRB in some listeners.

Podcast hosts seem to have a similar role for listeners as radio hosts. According to the study by Perks and Turner (2019), parasocial interactions and relationships with the host are among the central motives for using podcasts, so that listeners sometimes even listen to episodes whose topics they are not (initially) interested in. The PSR with the host is also expressed in the fact that the listeners contact him/her via social media, comment on pictures and write their own texts.

Two of the eight studies in the context of auditory media were concerned with music. According to the study by Kurtin et al. (2019), the strength of parasocial bonds with favorite musicians is explained by factors such as perceived physical attractiveness, social attractiveness, and competence. Similarly, an important factor in the development of parasocial bonds is the perceived authenticity of the musicians (as detected in the context of music casting shows by Ruth et al., 2016). Moreover, Kresovich’s (2022) study demonstrated that a strong PSR with a musician on the listener’s part can also affect the empathetic processing of the content of songs and the perceived personal connection to a song. Pop songs that address mental health issues, such as anxiety, depression, or suicidal thoughts, can thus have a particularly strong impact because they may increase the listener’s willingness to offer psychological support to others. However, in these studies, it was unclear through which media channels the PSR with favorite musicians was established. Even if the first contact with a musician was made via radio or a music streaming service, it is very likely that he or she was subsequently encountered more often in audiovisual media (television, YouTube, social media), which also explains why physical attractiveness is such a strong driver for PSR. If the contact had been purely auditory, the physical attractiveness of the musician would be a big unknown for the listener and could at best be imagined to be high. In other words, even if these two studies are included in the auditory superior category “radio, music, and podcasts” because the majority of music consumption is purely auditory, it is more likely that cross-media contacts with musicians explain the genesis of PSR, and thus also the effect of the music in purely auditory reception. This could be investigated in further studies.

Given the prominence of music in the lives of adolescents and young adults, there are very few studies that address parasocial phenomena and effects in music reception. Because listening to music plays a crucial role in the identity formation and development of adolescents (Dollase, 1997) and parasocial phenomena have been plausibly linked to identity formation and development (Schramm and Hartmann, 2007), connecting these two areas offers a new and generative perspective on research into the socializing and identity-building functions of listening to music.

#### 4.3.3 Parasocial phenomena in the use of film and television

A quarter of all studies conducted between 2016 and 2020 were carried out in the domain of film and television. The media offerings under scrutiny encompassed a broad spectrum of series (e.g., Holladay and Edgar, 2019; Jain et al., 2016; Leksmono, 2016; So and Shen, 2016; Sorlin, 2018) and movies (e.g., Driesmans et al., 2016; Hall, 2019, 2022; McDermott et al., 2018; Sheldon et al., 2021), extending to entertainment shows (Gabriel et al., 2018) and talent shows (Ruth et al., 2016; Shin, 2016), reality formats with advisory or self-help functions (Rasmussen and Ewoldsen, 2016), reports and news (Harwood et al., 2016; Landreville and Niles, 2019; Schartel Dunn, 2018; Sherman-Morris et al., 2020), documentaries (Bradshaw et al., 2020), and instructional videos (Beege et al., 2019). Even explicitly promotional forms, such as commercials (Phua, 2016), teleshopping (Lee and Park, 2017), and product placement (Carr, 2018; Dias et al., 2017), were sporadically represented in the PSI and PSR research into film and television, although their importance has decreased dramatically compared with studies conducted between 1956 and 2015 (see Liebers and Schramm, 2017a, 2019). Instead, such studies have shifted into the realm of social and new media, which provide much more effective advertising opportunities (see below).

When exploring the functions of favorite characters in films and on television, many studies have used established perspectives, particularly when the media characters are not predetermined by the study design. Therefore, questions have arisen regarding the role model and imitation functions of the admired media characters with whom a strong parasocial bond (PSR) has been formed (e.g., Newman, 2018; Sycoff and Cunningham, 2020), and the compensatory function of PSR when relationships in real life are lacking or inadequate. Individual deficits, such as attachment anxiety, have also been considered (e.g., Madison et al., 2016; Rosaen and Dibble, 2016). Under the paradigm of the Parasocial Contact Hypothesis (Schiappa et al., 2005), the extent to which parasocial contact with positively depicted members of outgroups improves attitudes and potential behavioral intentions towards these outgroups has also been investigated, with a focus on groups such as the lesbian–gay-bisexual–transexual (LGBT) community (Bond, 2021; Lissitsa and Kushnirovich, 2020; McDermott et al., 2018), asylum seekers and refugees (Schemer and Meltzer, 2020), and specific ethnicities (Cho et al., 2021; Harwood et al., 2016). In the context of PSRB, in addition to obvious research questions (e.g., “How does the cancellation of a favorite soap opera affect viewers’ habits and emotions?”; Natale, 2017), new scenarios that can lead to the termination or at least weakening of an existing PSR have also been examined. For example, Hu (2016) showed that viewers can experience a PSRB as a result of a scandal about their television star becoming public, and that the stronger the previous PSR with that star, the more intensely that PSRB is experienced.

Also, already known PSI-and PSR-promoting factors (cf. Liebers and Schramm, 2017a, pp. 43–45) on the part of media users and media personae are examined in new contexts, which contributes to a strengthening and validation of the previous findings. On the side of media users, for instance, gender (Greenwood et al., 2021; Hall, 2022; Hoewe et al., 2020) has been a significant factor in media contexts involving heroes and role models. When considering the compensatory functions of PSR, factors such as loneliness and attachment anxiety (Bernhold and Metzger, 2020; Rosaen and Dibble, 2016) or attachment style (Bernhold, 2019; Silver and Slater, 2019) have been identified as important, whereas in outgroup contexts, factors such as ethnocentrism (Hu et al., 2019) and homophobia (Bond, 2021) have been found to be significant. On the side of the media characters, factors such as perceived addressing and sociability (e.g., Beege et al., 2019; Cohen et al., 2019; Dibble et al., 2016; Shin, 2016), perceived attractiveness (Jain et al., 2016), likability (Rosaen and Dibble, 2017), perceived credibility and authenticity (Phua, 2016; Ruth et al., 2016), character traits (e.g., good vs. bad guy; Dias et al., 2017), and perceived similarity between the media character and media user (e.g., Phua, 2016; Rosaen and Dibble, 2017; Shen et al., 2018) have been identified as influential.

The context of film and television has also been examined to identify specific and novel facets of parasocial phenomena raising the question of whether these facets constitute distinct constructs, new forms of PSI and PSR, or overarching phenomena in which PSI and PSR may merge. Exemplary are the studies on narrative engagement (Schartel Dunn, 2018; Shen et al., 2018), retrospective imaginative involvement (Silver and Slater, 2019; Slater et al., 2018), and daydreaming conversations with personae (Madison and Porter, 2016). When these phenomena have been measured in addition to the relevant parasocial constructs, such as PSI and PSR, and correlated with them, strong correlations between these constructs have been detected. This is not surprising because the constructs already overlap at the theoretical level. However, such studies help us to continually question the definitions and explanatory powers of parasocial phenomena and distinguish them from related, overarching, and theoretically plausible constructs and phenomena. Subsequent studies that take this into account could further contribute to sharpening the findings.

In a few individual studies, new and innovative research questions have been raised, sometimes focusing on very specific, exciting, and highly controversial relationships. Tal-Or and Razpurker-Apfeld (2021), for example, investigated the effects of the congruence between the room temperature while watching a film and the mood of the media figures on PSR and viewer attitudes. Bozkurt and Hatipoglu (2017) found associations between Turkish TV series that ended (which may have led to many instances of PSRB and bad moods among viewers) and increased stock demand on the next trading day.

Longitudinal designs are rare in research into parasocial phenomena (Liebers and Schramm, 2017a, 2019). Although some studies published between 2016 and 2020 addressed parasocial phenomena in the context of long-term or repeated media use, such as binge-watching (Anghelcev et al., 2021; Nanda and Banerjee, 2020; Tukachinsky and Eyal, 2018), these have once again not been examined longitudinally with multiple waves of data collection. This means that the dynamic development of parasocial phenomena and their associated effects is still insufficiently explained.

#### 4.3.4 Parasocial phenomena in the use of social and new media

Of the 112 studies that examined parasocial phenomena in the use of social and new media, only a few considered digital assets other than social media, such as computer games (e.g., Banks and Bowman, 2016), online trading platforms (e.g., Alizadeh, 2019), or various streaming platforms for videos, video games, or live events (e.g., Hu et al., 2017; Lee et al., 2021; Lim et al., 2020). The majority of studies focused on individual social media platforms, including above all *Twitter* (e.g., Dai and Walther, 2018; Kim and Song, 2016), *Facebook* (e.g., Ledbetter and Redd, 2016; Wellman, 2021), *Instagram* (e.g., Blight et al., 2017; Breves et al., 2019), and *YouTube* (e.g., Rihl and Wegener, 2019; Tolbert and Drogos, 2019), or even considered an aggregated view of all popular platforms in cases where social media were not constrained or predefined, such as when inquiries were made about a favorite media character across all social media platforms.

One central research focus in the field of social media revolved around the fundamental question of what factors explain the strength of PSI and PSR with favorite media characters—mostly influencers—on social media platforms. Here, factors identical to those involved in film and television come into play, such as perceived similarity to the influencer (e.g., Hu et al., 2020; Tolbert and Drogos, 2019), attractiveness (e.g., Lee and Watkins, 2016), competence or trustworthiness (e.g., de Jans et al., 2018; Lou and Kim, 2019), and credibility (e.g., de Jans et al., 2018; Ledbetter and Redd, 2016), or other factors that arise when using interactive media, such as experiencing genuine social interactions: Adolescents who have, for instance, experienced social interactions with their favorite media characters on *Twitter*, in the form of retweets or replies to tweets, develop stronger PSR than those who have not experienced such interactions (Bond, 2016). The social presence of influencers, which can result from such social interactions, appears to be a higher-level factor that can mediate the strength of parasocial phenomena in social media (Ledbetter and Meisner, 2021). Moreover, factors such as sharing information and the resulting sense of community with both influencers and other social media users are mentioned as PSI-and PSR-promoting factors (Blight et al., 2017). An interesting question that should be explored both conceptually and empirically in future studies is whether the term “parasocial phenomena” is still applicable in such cases where media-mediated social phenomena are the primary aspects (see Hartmann, 2023).

In the context of film and television, advertising-related questions in PSI and PSR research have almost disappeared in recent years. However, they remain dominant in the context of social media. Dozens of studies have investigated the extent to which and under what conditions strong PSR with influencers increases the effectiveness of advertising communication. A crucial factor seems to be the existence of a certain fit between the influencer and the advertised product, because influencer–product congruence plays a significant role in shaping the perception of product recommendations as both competent and trustworthy (e.g., Gong and Li, 2017). However, studies have also demonstrated that long-term PSR with an influencer can obscure a lack of fit with the product, resulting in the uncritical and unquestioning acceptance by their fans of products directly or indirectly promoted by highly popular influencers (Breves et al., 2019). Preliminary findings suggest that this effect is particularly prominent when products are implicitly presented and promoted, and the sales intention is not particularly obvious (Choi and Lee, 2019). This even seems to work with children who have strong PSR towards admired influencers even when the selling intention is disclosed (Boerman and van Reijmersdal, 2020). In general, the level of an individual’s persuasion knowledge is also a crucial factor, and those with more knowledge quickly recognize advertising strategies and show less susceptibility to their influence. Those who show susceptibility not only develop a more positive attitude towards the advertised brand and a stronger intention to purchase, but also—and this is another dimension of effect within the context of social media—more frequently recommend the product to others through electronic word of mouth (Hwang and Zhang, 2018).

The impact of influencers has also been investigated in other persuasive contexts, such as health communication. It has been demonstrated that credible and authentic-seeming influencers can use persuasive social influence to increase the self-efficacy of their followers in seeking help for depression (Lee et al., 2021) or engaging in a diet in the case of overweight individuals (Phua and Tinkham, 2016). PSR towards prominent individuals who maintain contact with their fans through social media can also contribute to greater understanding and less stigmatization when these individuals admit to suffering mental illnesses (Parrott et al., 2020). Parasocial phenomena associated with the use of social media have also been investigated in the political context, such as the potential impact of tweets from Donald Trump (McDonnell and Wheeler, 2019). In a cross-media study, a positive relationship was detected between PSR towards Donald Trump and voting for him in the 2016 election, supporting his policies and media presence, and following his tweets on social media (Cohen and Holbert, 2021). This sociopolitical area is highly relevant because an increasing number of politicians perceive themselves as influencers, and, being aware of social media’s potent impact, strategically incorporate those platforms into their communications. Given recent political events and uncertainties, this research area is expected to become increasingly important in coming years. Other recent publications, which were not included in our sample, have shown indications of this trend (e.g., Schmuck et al., 2022).

#### 4.3.5 Parasocial phenomena in cross-media use

Only a few of the 77 studies in the “cross-media” category specialized in explaining parasocial phenomena that have arisen through cross-media use. Many of these studies examined parasocial relationships (PSR) with celebrities, stars, or favorite media characters and the usual factors that explain the strength of these cross-media PSRs independently of specific media (e.g., Bond, 2018; Bui, 2017; Gleason et al., 2017; Jennings and Alper, 2016; Richards and Calvert, 2016; Stever, 2017). Of particular interest is studies that aim to explain the genesis and effects of parasocial phenomena based on a specific cross-media usage pattern, or that are designed to empirically demonstrate the explanatory contributions of different media to parasocial phenomena. Exemplary research questions in this area include the following: To what extent does viewing reality television programs and following their *Twitter* profiles influence the misuse of illicit drugs and prescription medication among university students? (Fogel and Shlivko, 2016). To what extent do interactions with a female series protagonist on *Facebook* beyond the series itself lead to parasocial interaction? (Kyewski et al., 2018). To what extent does the use of *Twitter* affect the reception of Islamic reality shows? (Shariffadeen and Manaf, 2017). To what extent does the type of contact influence prejudices towards immigrants and the attribution of humanity, and how does this differ between news media and entertainment formats? (Visintin et al., 2017).

A further subgroup of cross-media studies has scrutinized parasocial phenomena that originate in one medium (e.g., television) but are communicated in a different medium (e.g., social media). For example, Daniel and Westerman (2017) conducted a content analysis of nearly 1,000 tweets with the hashtag #jonsnow after Jon Snow, a central and popular media character in the series “Game of Thrones,” died, and millions of fans had to deal with PSRB. Similar designs have been used in the studies of Wilson and Ertan (2020), Foss (2020), Hornsby and Groover (2020), and Kretz (2020).

Future cross-media studies could more closely examine how various media (offerings) contribute cumulatively, complementarily, or even substitutively to the genesis and shaping of parasocial phenomena, in what configurations and under what conditions. This would ideally involve longitudinal empirical tracing, so there are enormous methodological challenges associated with this approach.

## 5 Conclusion

### 5.1 Overall interpretation

When Donald Horton and R. Richard Wohl set the ball rolling for research into parasocial phenomena in 1956, neither of them could have imagined that more than 65 years later their idea of the interaction and relationship between recipients and media characters would be the starting point for over 520 empirical studies. In the course of these studies and with the help of numerous theoretical works, the concept has been refined many times, made measurable, transferred to other media objects, its causes examined on the recipient and media character side and the various consequences of parasocial phenomena investigated. The last 65 years have made it possible to better understand the concept of parasocial phenomena and to examine it in a more differentiated way, and have also provided insight into the potential variety of contexts in which parasocial phenomena play a role. At present, we know more about parasocial phenomena than about many other central concepts of media reception and media effects research. The fact that more studies were produced in the years 2016 to 2020 alone than in the previous 60 years speaks for the great attractiveness, dynamism and differentiation of this field of research. The main parasocial phenomena currently being researched in the various media and how this is to be interpreted was already the subject of the previous section and will not be summarized again here.

It can be noted and is of course not surprising that PSI and PSR seem to be a—if not the—key concept for the use and effect of social media, but for this reason it is also used very arbitrarily and without reflection, because many of the phenomena studied often do not involve parasocial communication at all, but rather communication mediated by media. In the same way that social media has gained in importance in research into parasocial phenomena, all cross-media usage constellations in which social media is integrated have naturally also gained in importance—and this applies to almost everyone, because who uses traditional mass media exclusively without making use of social media in parallel or at least in addition to it? What remains the same as in the last 65 years: PSI and PSR research remains research primarily on audio-visual media; purely auditory media or print media are only examined very selectively, but then usually always very profitably.

A central point that comes up again and again in the discussion about research into parasocial phenomena and also became clear in our analysis is the measurement of the various parasocial phenomena. This not only reflects the heterogeneity of the methodological approaches of the studies but also the ongoing lack of differentiation of parasocial phenomena. An apt example is the PSI-Scale by Rubin et al. (1985). It’s name indicates that it can be used to measure PSI. This is contradicted by the individual items of the scale, most of which measure PSR (Dibble et al., 2023). The lack of definitional clarity is particularly concerning when we consider that the PSI-Scale is still by far the most widely used measuring instrument for parasocial phenomena. This imprecision in measuring parasocial phenomena is further exacerbated by a ubiquitous lack of agreement on the dimensions of parasocial phenomena. Therefore, it is not surprising that some studies still develop their own scales (e.g., Banks and Bowman, 2016; Erickson and Dal Cin, 2018; Richards and Calvert, 2017; Song and Fox, 2016).

In addition to the measurement of parasocial phenomena, the methodological approach can also be critically interpreted. A significant proportion of findings continues to rely on survey data collected with correlational research designs. Here, theoretical considerations or plausibilities are usually employed to infer causal relationships and directions. Therefore, more investigation of parasocial phenomena and their interrelationships with other factors in experimental settings is desirable, and has already become more important in recent years.

### 5.2 Limitations

Our review and analysis are of course subject to some limitations. For example, it cannot be ruled out that we missed a few studies despite very intensive and duplicate searches in three databases using several search terms. Furthermore, we only have English and German-language publications in our sample—there will certainly have been a few relevant studies in French, Spanish or one of the many Asian languages, for example, about which we cannot make any statements. Although about 80% of the sample consists of journal articles from ranked journals, we also have studies from very specialized and unranked journals, so it cannot be assumed that the study quality is always consistently high. Finally, due to the descriptive and, in terms of perspectives and key findings, highlight-like nature of our review (which is unavoidable with 281 studies), we cannot derive any overarching empirical findings, as meta-analyses and systematic reviews could. However, this was not the aim of our analysis.

### 5.3 Outlook

Finally, we want to give an outlook on possible future research on parasocial phenomena, which would close particularly large gaps in current research. Let us start with the measurement problem mentioned above. It is not always necessary to reinvent the wheel, as there are now several established measurement tools that can be used in numerous contexts. It would be useful to compare existing instruments to identify exactly what each instrument measures (Dibble et al., 2016, 2023). Nevertheless, suitable measuring instruments are still not available for many investigation contexts, and compiling items from different scales remains a common approach (e.g., Hu, 2016). Uniform consensus on the definitions and dimensions of constructs is essential for the approximate comparability of the empirical findings of parasocial research and poses an ongoing challenge for future research.

It should be noted that nearly all studies on parasocial phenomena were cross-sectional in design (with few exceptions). Although exciting snapshots can be taken in this way, knowledge about the dynamic development of parasocial phenomena and their interactions with other influencing factors over time cannot be gained with such studies. Questions such as “Does the relevance of the physical attractiveness of a media character decrease over time in PSR?” or “Do PSRs weaken when the viewer finds a new real friend?” cannot yet be answered. The long-term perspective of how PSR and PSRB can functionally shape and develop for people over the course of a long time, and especially in old age, would be of greatest interest and social relevance. Despite the relatively high methodological effort required for such longitudinal studies, the knowledge gained is essential for a comprehensive understanding of parasocial phenomena (see recent studies published after 2020, such as Liebers, 2021; Siegenthaler et al., 2023).

Moreover, samples must become more heterogeneous than has been the case thus far. Studies should move away from a sole reliance on easily accessible student samples with a disproportionate representation of women, towards the utilization of more diverse samples that include larger proportions of men, individuals with lower levels of education, and older individuals.

The assumption that parasocial phenomena are primarily benign and that the user–media character constellation typically consists of an intriguing and admirable media character and a fascinated and admiring media user, still appears to be too widespread. Only five studies between 2016 and 2020 dealt with negative parasocial phenomena. In accordance with the basic idea that parasocial processes are very similar to real social processes in many ways, a range of parasocial phenomena that correspond to the range of social phenomena in the real world should be investigated in the future. The first steps have already been taken by also focusing on antiheroes, villains, and questionable or morally ambiguous media characters (Bonus et al., 2021; Greenwood et al., 2021; Möri et al., 2023; Oliver et al., 2019; Sorlin, 2018). However, we should not only examine the fascination and thus the positive PSRs with these media characters, but the potentially negative parasocial phenomena, which could be associated with affective components, such as dislike, contempt, and hatred (Bernhold, 2019). After all, in the real world, people more often despise and hate a villain than admire and love him. Another step towards understanding the negative facets of parasocial phenomena has been taken with the occasional focus on the strongly negative characteristics of recipients, such as the Dark Triad. While these traits can also explain positive parasocial phenomena, for example the need for romance (cf. Liebers and Schramm, 2022), they could possibly explain even better why people are attracted to those media characters who are inferior to them and appear submissive. In short, research on parasocial phenomena could take a greater interest in socially dysfunctional phenomena that can be functional for certain individuals.

Cultural differences represent an understudied aspect of parasocial processes. Just as interpersonal interactions and the development of relationships differ between collectivist and individualistic cultures, parasocial processes may also differ. Although several studies have investigated intercultural effects (e.g., Ramasubramanian and Kornfield, 2012), only a few have compared different cultures and collected the corresponding data (e.g., Schmid and Klimmt, 2011). These studies have already identified cultural differences, so comparing cultures in future studies should be meaningful and necessary.

The comparison of different media is as central to parasocial research as the comparison of different cultures. The question of whether parasocial phenomena develop in media that allow audiovisual interactions with a media character in the same way as they do in media that only allow auditory contact can only be partly answered at this time. Does the physical attractiveness of a podcast host play as important a role in PSR with him/her, as it does with an influencer? Is the experience of presence a more central factor for PSI when reading a book than in virtual environments, where a sense of presence is not as natural? Can parasocial phenomena with radio or book characters become as intense as with television or social media characters? The general concentration on parasocial phenomena in the audiovisual context of social media and in film and television is strongly related to the neglect of media comparisons. A greater focus on the previously neglected peculiarities of media contexts and comparisons of different media could be fruitful and contribute to a holistic understanding of parasocial phenomena. The desiderata highlighted here represent some of the research options that could become relevant and interesting in the coming years.
