# Supplementary material for: Research trends on parasocial interactions and relationships with media characters. A review of 281 English and German-language studies from 2016 to 2020
Source: Front Psychol. 2024 Sep 26;15:1418564. doi: 10.3389/fpsyg.2024.1418564 (PMC11464444; doi:10.3389/fpsyg.2024.1418564)
Supplement: Supplementary file 1 [file Table_1.pdf]

Appendix 1: References of the 281 Studies

* Book Chapter

\+ German language

Abrams, J. R., McGaughey, K. J., & Haghighat, H. (2018). Attitudes toward Muslims: a test of the parasocial contact hypothesis and contact theory. *Journal of Intercultural Communication Research*, *47*, 276–292. https://doi.org/10.1080/17475759.2018.1443968

Adam, A. (2019). Perceptions of infidelity: A comparison of sexual, emotional, cyber-, and parasocial behaviors. *Interpersona*, *13*, 237–252. https://doi.org/10.5964/ijpr.v13i2.376

Agnihotri, A., & Bhattacharya, S. (2021). Endorsement effectiveness of celebrities versus social media influencers in the materialistic cultural environment of India. *Journal of International Consumer Marketing*, *33*, 280–302. https://doi.org/10.1080/08961530.2020.1786875

Aguiar, N. R., Richards, M. N., Bond, B. J., Brunick, K. L., & Calvert, S. L. (2019). Parents' perceptions of their children's parasocial relationships: The recontact study. *Imagination, Cognition and Personality*, *38*, 221–249. https://doi.org/10.1177/0276236618771537

Aguiar, N. R., Richards, M. N., Bond, B. J., Putnam, M. M., & Calvert, S. L. (2019). Children's parasocial breakups with media characters from the perspective of the parent. *Imagination, Cognition and Personality*, *38*, 193–220. https://doi.org/10.1177/0276236618809902

Alizadeh, H. (2019). Analysis of the effect of para-social interactions on impulse buying behavior in social commerce: Case study of baby clothing stores. *Asian Journal of Management Sciences & Education*, *8*, 113–122.

Anghelcev, G., Sar, S., Martin, J. D., & Moultrie, J. L. (2021). Binge-watching serial video content: Exploring the subjective phenomenology of the binge-watching experience. *Mass Communication and Society*, *24*, 130–154. https://doi.org/10.1080/15205436.2020.1811346

Aw, E. C.-X., & Labrecque, L. I. (2020). Celebrity endorsement in social media contexts: understanding the role of parasocial interactions and the need to belong. *Journal of Consumer Marketing*, *37*, 895–908. https://doi.org/10.1108/JCM-10-2019-3474

Banks, J., & Bowman, N. D. (2016a). Avatars are (sometimes) people too: Linguistic indicators of parasocial and social ties in player-avatar relationships. *New Media & Society*, *18*, 1257–1276. https://doi.org/10.1177/1461444814554898

Banks, J., & Bowman, N. D. (2016b). Emotion, anthropomorphism, realism, control: Validation of a merged metric for player-avatar interaction (PAX). *Computers in Human Behavior*, *54*, 215–223. https://doi.org/10.1016/j.chb.2015.07.030

Beege, M., Nebel, S., Schneider, S., & Rey, G. D. (2019). Social entities in educational videos: Combining the effects of addressing and professionalism. *Computers in Human Behavior*, *93*, 40–52. https://doi.org/10.1016/j.chb.2018.11.051

Beege, M., Ninaus, M., Schneider, S., Nebel, S., Schlemmel, J., Weidenmüller, J. et al. (2020). Investigating the effects of beat and deictic gestures of a lecturer in educational videos. *Computers & Education*, *156*, 103955. https://doi.org/10.1016/j.compedu.2020.103955

Beege, M., Schneider, S., Nebel, S., & Rey, G. D. (2017). Look into my eyes! Exploring the effect of addressing in educational videos. *Learning and Instruction*, *49*, 113–120. https://doi.org/10.1016/j.learninstruc.2017.01.004

Behm-Morawitz, E., Aubrey, J. S., Pennell, H., & Kim, K. B. (2019). Examining the effects of MTV's 16 and pregnant on adolescent girls' sexual health: The implications of character affinity, pregnancy risk factors, and health literacy on message effectiveness. *Health Communication*, *34*, 180–190. https://doi.org/10.1080/10410236.2017.1399506

Bérail, P. de, Guillon, M., & Bungener, C. (2019). The relations between YouTube addiction, social anxiety and parasocial relationships with YouTubers: A moderated-mediation model based on a cognitive-behavioral framework. *Computers in Human Behavior*, *99*, 190–204. https://doi.org/10.1016/j.chb.2019.05.007

Bernhold, Q. S. (2019). Parasocial relationships with disliked television characters, depressive symptoms, and loneliness among older adults. *Journal of Applied Communication Research*, *47*, 548–570. https://doi.org/10.1080/00909882.2019.1679384

Bernhold, Q. S., & Metzger, M. (2020). Older adults' parasocial relationships with favorite television characters and depressive symptoms. *Health Communication*, *35*, 168–179. https://doi.org/10.1080/10410236.2018.1548336

Bingaman, J. (2020). "Dude I've never felt this way towards a celebrity death": Parasocial grieving and the collective mourning of Kobe Bryant on Reddit. *Omega - Journal of Death and Dying*. https://doi.org/10.1177/0030222820971531

Blight, M. G., Ruppel, E. K., & Schoenbauer, K. V. (2017). Sense of community on Twitter and Instagram: Exploring the roles of motives and parasocial relationships. *Cyberpsychology, Behavior and Social Networking*, *20*, 314–319. https://doi.org/10.1089/cyber.2016.0505

Boehmer, J. (2016). Does the game really change? How students consume mediated sports in the age of social media. *Communication & Sport*, *4*, 460–483. https://doi.org/10.1177/2167479515595500

Boerman, S. C. (2020). The effects of the standardized instagram disclosure for micro- and meso-influencers. *Computers in Human Behavior*, *103*, 199–207. https://doi.org/10.1016/j.chb.2019.09.015

Boerman, S. C., & van Reijmersdal, E. A. (2020). Disclosing influencer marketing on YouTube to children: The moderating role of para-social relationship. *Frontiers in Psychology*, *10*, 3042. https://doi.org/10.3389/fpsyg.2019.03042

Bond, B. J. (2016). Following your "friend": Social media and the strength of adolescents' parasocial relationships with media personae. *Cyberpsychology, Behavior and Social Networking*, *19*, 656–660. https://doi.org/10.1089/cyber.2016.0355

Bond, B. J. (2018). Parasocial relationships with media personae: Why they matter and how they differ among heterosexual, lesbian, gay, and bisexual adolescents. *Media Psychology*, *21*, 457–485. https://doi.org/10.1080/15213269.2017.1416295

Bond, B. J. (2021). The development and influence of parasocial relationships with television characters: A longitudinal experimental test of prejudice reduction through parasocial contact. *Communication Research*, *48*, 573-593. https://doi.org/10.1177/0093650219900632

Bonus, J. A., Matthews, N. L., & Wulf, T. (2021). The impact of moral expectancy violations on audiences' parasocial relationships with movie heroes and villains. *Communication Research*, *48*, 550–572. https://doi.org/10.1177/0093650219886516

Bostwick, E. N., & Lookadoo, K. L. (2017). The return of the king: How Cleveland reunited with LeBron James after a parasocial breakup. *Communication & Sport*, *5*, 689–711. https://doi.org/10.1177/2167479516659460

Bozkurt, I., & Hatipoglu, M. (2017). The relation between parasocial breakup and investor behaviours. *Journal of Academic Research in Acounting, Finance and Management Sciences*, *7*, 87–96.

Bradshaw, A. S., Treise, D., Shelton, S. S., Cretul, M., Raisa, A., Bajalia, A. et al. (2020). Propagandizing anti-vaccination: Analysis of Vaccines Revealed documentary series. *Vaccine*, *38*, 2058–2069. https://doi.org/10.1016/j.vaccine.2019.12.027

Breves, P. L., Liebers, N., Abt, M., & Kunze, A. (2019). The perceived fit between Instagram influencers and the endorsed brand. *Journal of Advertising Research*, *59*, 440–454. https://doi.org/10.2501/JAR-2019-030

Brodie, Z., & Ingram, J. (2021). The dark triad of personality and hero/villain status as predictors of parasocial relationships with comic book characters. *Psychology of Popular Media*, *10*, 230–242. https://doi.org/10.1037/ppm0000323

Bui, N. H. (2017). Exploring similarity characteristics, identification and parasocial interactions in choice of celebrities. *Psychology of Popular Media Culture*, *6*, 21–31. https://doi.org/10.1037/ppm0000082

Burnasheva, R., & Suh, Y. G. (2022). The moderating role of parasocial relationships in the associations between celebrity endorser's credibility and emotion-based responses. *Journal of Marketing Communications*, *28*, 343–359. https://doi.org/10.1080/13527266.2020.1862894

Calvert, S. L., Putnam, M. M., Aguiar, N. R., Ryan, R. M., Wright, C. A., Liu, Y. H. A. et al. (2020). Young children's mathematical learning from intelligent characters. *Child Development*, *91*, 1491–1508. https://doi.org/10.1111/cdev.13341

Carr, D. J. (2018). Exploring the role of parasocial relationships on product placement effectiveness. *Amercian Communication Journal*, *20*, 31–45.

Centeno, D. G. (2016). Celebrities' parasocial interaction and relationship: Predictor of voting preference towards endorsed political candidates. *Philippine Management Review*, *23*, 53–68.

*Chang, E.-C., & Woo, T. C.-T. (2020). Follow me!: How internet celebrities in China (Wanghong) attract and influence their Chinese fans. In C. L. Wang (Ed.), *Handbook of Research on the Impact of Fandom in Society and Consumerism* (pp. 397–421). IGI Global.

Chen, C.-P. (2016). Forming digital self and parasocial relationships on YouTube. *Journal of Consumer Culture*, *16*, 232–254. https://doi.org/10.1177/1469540514521081

Cho, H., Li, W., Cannon, J., Lopez, R., & Song, C. C. (2021). Testing three explanations for stigmatization of people of Asian descent during COVID-19: maladaptive coping, biased media use, or racial prejudice? *Ethnicity & Health*, *26*, 94–109. https://doi.org/10.1080/13557858.2020.1830035

Choi, S., Kim, I., Cha, K., Suh, Y.-K., & Kim, K.-H. (2019). Travelers' parasocial interactions in online travel communities. *Journal of Travel & Tourism Marketing*, *36*, 888–904. https://doi.org/10.1080/10548408.2019.1657053

Choi, W., & Lee, Y. (2019). Effects of fashion vlogger attributes on product attitude and content sharing. *Fashion and Textiles*, *6*. https://doi.org/10.1186/s40691-018-0161-1

Chung, S., & Cho, H. (2017). Fostering parasocial relationships with celebrities on social media: Implications for celebrity endorsement. *Psychology & Marketing*, *34*, 481–495. https://doi.org/10.1002/mar.21001

Cohen, E. L., & Hoffner, C. (2016). Finding meaning in a celebrity's death: The relationship between parasocial attachment, grief, and sharing educational health information related to Robin Williams on social network sites. *Computers in Human Behavior*, *65*, 643–650. https://doi.org/10.1016/j.chb.2016.06.042

Cohen, E. L., Myrick, J. G., & Hoffner, C. A. (2021). The effects of celebrity silence breakers: Liking and parasocial relationship strength interact to predict the social influence of celebrities' sexual harassment allegations. *Mass Communication and Society*, *24*, 288–313. https://doi.org/10.1080/15205436.2020.1839102

Cohen, E. L., & Tyler, W. J. (2016). Examining perceived distance and personal authenticity as mediators of the effects of ghost-tweeting on parasocial interaction. *Cyberpsychology, Behavior and Social Networking*, *19*, 342–346. https://doi.org/10.1089/cyber.2015.0657

Cohen, J., & Hershman-Shitrit, M. (2017). Mediated relationships with TV characters. The effects of perceived and actual similarity in personality traits. *Scientific Study of Literature*, *7*, 109–128. https://doi.org/10.1075/ssol.7.1.05coh

Cohen, J., & Holbert, R. L. (2021). Assessing the predictive value of parasocial relationship intensity in a political context. *Communication Research*, *48*, 501–526. https://doi.org/10.1177/0093650218759446

Cohen, J., Oliver, M. B., & Bilandzic, H. (2019). The differential effects of direct address on parasocial experience and identification: Empirical evidence for conceptual difference. *Communication Research Reports*, *36*, 78–83. https://doi.org/10.1080/08824096.2018.1530977

Dai, Y., & Walther, J. B. (2018). Vicariously experiencing parasocial intimacy with public figures through observations of interactions on social media. *Human Communication Research*, *44*, 322–342. https://doi.org/10.1093/hcr/hqy003

Daniel, E. S., Crawford Jackson, E. C., & Westerman, D. K. (2018). The influence of social media influencers: Understanding online vaping communities and parasocial interaction through the lens of Taylor's six-segment strategy wheel. *Journal of Interactive Advertising*, *18*, 96–109. https://doi.org/10.1080/15252019.2018.1488637

Daniel, E. S., & Westerman, D. K. (2017). Valar Morghulis (all parasocial men must die): Having nonfictional responses to a fictional character. *Communication Research Reports*, *34*, 143–152. https://doi.org/10.1080/08824096.2017.1285757

David, K., Myers, M. E., Perry, S. D., Gouse, V., & Stein, C. B. (2019). Examination of insecure attachment and the potential for parasocial parental attachment (PPA) to a favorite celebrity through attachment theory. *North American Journal of Psychology*, *21*, 387–406.

DeGroot, J. M., & Leith, A. P. (2018). R.I.P. Kutner: Parasocial grief following the death of a television character. *Omega - Journal of Death and Dying*, *77*, 199–216. https://doi.org/10.1177/0030222815600450

Derrick, J. L., Keefer, L. A., & Troisi, J. D. (2019). Who needs friends? Personality as a predictor of social surrogate use. *Personality and Individual Differences*, *138*, 349–354. https://doi.org/10.1016/j.paid.2018.10.028

Dias, J. A., Dias, J. G., & Lages, C. (2017). Can negative characters in soap operas be positive for product placement? *Journal of Business Research*, *71*, 125–132. https://doi.org/10.1016/j.jbusres.2016.10.010

Dibble, J. L., Hartmann, T., & Rosaen, S. F. (2016). Parasocial interaction and parasocial relationship: Conceptual clarification and a critical assessment of measures. *Human Communication Research*, *42*, 21–44. https://doi.org/10.1111/hcre.12063

Ding, Y., & Qiu, L. (2017). The impact of celebrity-following activities on endorsement effectiveness on microblogging platforms. *Nankai Business Review International*, *8*, 158–173. https://doi.org/10.1108/NBRI-11-2016-0043

Driesmans, K., Vandenbosch, L., & Eggermont, S. (2016). True love lasts forever: The influence of a popular teenage movie on Belgian girls' romantic beliefs. *Journal of Children and Media*, *10*, 304–320. https://doi.org/10.1080/17482798.2016.1157501

Drizin, J. H., Malcarne, V. L., Schiaffino, M. K., & Wells, K. J. (2018). College Women's Responses to a Celebrity Health Disclosure. *Health Communication*, *33*, 1302–1307. https://doi.org/10.1080/10410236.2017.1351276

El Damanhoury, K. (2020). Entertainment-education versus extremism: Examining parasocial interaction among Arab viewers of anti-ISIS TV drama. *Journal of Deradicalization*, *24*, 40–78.

Ellithorpe, M. E., & Brookes, S. E. (2018). I didn't see that coming: Spoilers, fan theories, and their influence on enjoyment and parasocial breakup distress during a series finale. *Psychology of Popular Media Culture*, *7*, 250–263. https://doi.org/10.1037/ppm0000134

Erickson, S. E., & Dal Cin, S. (2018). Romantic parasocial attachments and the development of romantic scripts, schemas and beliefs among adolescents. *Media Psychology*, *21*, 111–136. https://doi.org/10.1080/15213269.2017.1305281

Eriksson, G. (2016). The 'ordinary-ization' of televised cooking expertise: A historical study of cooking instruction programmes on Swedish television. *Discourse, Context & Media*, *13*, 29–39. https://doi.org/10.1016/j.dcm.2016.01.001

Ertan, C., & Wilson, S. (2020). The presentation of hegemonic masculinity, parasocial interaction and transnational online communities: A case study of the Turkish series "Erkenci Kuş". *Mediterranean Journal of Humanities*, *10*, 483–498. https://doi.org/10.13114/MJH.2020.544

Escalas Edson, J., & Bettman, J. R. (2017). Connecting with celebrities: How consumers appropriate celebrity meanings for a sense of belonging. *Journal of Advertising*, *46*, 297–308. https://doi.org/10.1080/00913367.2016.1274925

*+Eschenauer, E., Junggeburth, J., & Knospe, M. (2017). LeFloid - Meinungsführer im Netz? Eine Untersuchung zur Beziehung von Rezipient und YouTube-Star [LeFloid - opinion leader on the net? An investigation into the relationship between recipient and YouTube star]. In J. Echterbruch, J. Geuecke, N. Jungmann, & T. Schnödewind (Eds.), *Populismus, Terror und Wahlentscheidungen in Alten und Neuen Medien [Populism, terror and voting decisions in old and new media]* (pp. 135–156). Frank & Timme.

Esteban-Santos, L., García Medina, I., Carey, L., & Bellido-Pérez, E. (2018). Fashion bloggers: communication tools for the fashion industry. *Journal of Fashion Marketing and Management*, *22*, 420–437. https://doi.org/10.1108/JFMM-10-2017-0101

Farivar, S., Wang, F., & Yuan, Y. (2021). Opinion leadership vs. para-social relationship: Key factors in influencer marketing. *Journal of Retailing and Consumer Services*, *59*, 102371. https://doi.org/10.1016/j.jretconser.2020.102371

Ferchaud, A., & Oliver, M. B. (2019). It's my choice: The effects of moral decision-making on narrative game engagement. *Journal of Gaming & Virtual Worlds*, *11*, 101–118. https://doi.org/10.1386/jgvw.11.2.101_1

Finsterwalder, J., Yee, T., & Tombs, A. (2017). Would you forgive Kristen Stewart or Tiger Woods or maybe Lance Armstrong? Exploring consumers' forgiveness of celebrities' transgressions. *Journal of Marketing Management*, *33*, 1204–1229. https://doi.org/10.1080/0267257X.2017.1382553

Fogel, J., & Shlivko, A. (2016). Reality television programs are associated with illegal drug use and prescription drug misuse among college students. *Substance Use & Misuse*, *51*, 62–72. https://doi.org/10.3109/10826084.2015.1082593

Foss, K. A. (2020). Death of the slow-cooker or #crock-potsinnocent? This Is Us , parasocial grief, and the crock-pot crisis. *Journal of Communication Inquiry*, *44*, 69–89. https://doi.org/10.1177/0196859919826534

Foss, K. A., & Blake, K. (2019). "It's natural and healthy, but I don't want to see it": Using entertainment-education to improve attitudes toward breastfeeding in public. *Health Communication*, *34*, 919–930. https://doi.org/10.1080/10410236.2018.1440506

Frolich, M. A., Aguiar, N. R., Putnam, M. M., & Calvert, S. L. (2020). Adult reports of Pokémon GO play: Stronger parasocial relationships predict increased nostalgia and decreased app play. *Imagination, Cognition and Personality*, *39*, 261–276. https://doi.org/10.1177/0276236618822786

Fu, S., Xu, Y., & Yan, Q. (2019). Enhancing the parasocial interaction relationship between consumers through similarity effects in the context of social commerce. *Journal of Strategic Marketing*, *27*, 100–118. https://doi.org/10.1080/0965254X.2017.1384045

Gabriel, S., Paravati, E., Green, M. C., & Flomsbee, J. (2018). From apprentice to president: The role of parasocial connection in the election of Donald Trump. *Social Psychological and Personality Science*, *9*, 299–307. https://doi.org/10.1177/1948550617722835

Gil-Egui, G., Kern-Stone, R., & Forman, A. E. (2017). Till death do us part? Conversations with deceased celebrities through memorial pages on Facebook. *Celebrity Studies*, *8*, 262–277. https://doi.org/10.1080/19392397.2016.1259076

Gleason, T. R., Theran, S. A., & Newberg, E. M. (2017). Parasocial interactions and relationships in early adolescence. *Frontiers in Psychology*, *8*. https://doi.org/10.3389/fpsyg.2017.00255

Gleason, T. R., Theran, S. A., & Newberg, E. M. (2020). Connections between adolescents' parasocial interactions and recollections of childhood imaginative activities. *Imagination, Cognition and Personality*, *39*, 241–260. https://doi.org/10.1177/0276236619825810

Gong, W. (2021). Effects of parasocial interaction, brand credibility and product involvement on celebrity endorsement on microblog. *Asia Pacific Journal of Marketing and Logistics*, *33*, 1437–1454. https://doi.org/10.1108/APJML-12-2019-0747

Gong, W., & Li, X. (2017). Engaging fans on microblog: the synthetic influence of parasocial interaction and source characteristics on celebrity endorsement. *Psychology & Marketing*, *34*, 720–732. https://doi.org/10.1002/mar.21018

Gong, W., & Li, X. (2019). Microblogging reactions to celebrity endorsement: effects of parasocial relationship and source factors. *Chinese Journal of Communication*, *12*, 185–203. https://doi.org/10.1080/17544750.2018.1511607

Greenwood, D., McCutcheon, L. E., Collisson, B., & Wong, M. (2018). What's fame got to do with it? Clarifying links among celebrity attitudes, fame appeal, and narcissistic subtypes. *Personality and Individual Differences*, *131*, 238–243. https://doi.org/10.1016/j.paid.2018.04.032

Greenwood, D., Ribieras, A., & Clifton, A. (2021). The dark side of antiheroes: Antisocial tendencies and affinity for morally ambiguous characters. *Psychology of Popular Media*, *10*, 165–177. https://doi.org/10.1037/ppm0000334

Gregg, P. B. (2018). Parasocial breakup and Twitter: The firing of Barb Abney. *Journal of Broadcasting & Electronic Media*, *62*, 38–50. https://doi.org/10.1080/08838151.2017.1402900

Hall, A. E. (2019). Identification and parasocial relationships with characters from Star Wars: The force awakens. *Psychology of Popular Media Culture*, *8*, 88–98. https://doi.org/10.1037/ppm0000160

Hall, A. E. (2020). Audience responses to diverse superheroes: The roles of gender and race in forging connections with media characters in superhero franchise films. *Psychology of Aesthetics, Creativity, and the Arts*. https://doi.org/10.1037/aca0000363

Handarkho, Y. D. (2020). Impact of social experience on customer purchase decision in the social commerce context. *Journal of Systems and Information Technology*, *22*, 47–71. https://doi.org/10.1108/JSIT-05-2019-0088

Handarkho, Y. D. (2021). Understanding mobile payment continuance usage in physical store through social impact theory and trust transfer. *Asia Pacific Journal of Marketing and Logistics*, *33*, 1071–1087. https://doi.org/10.1108/APJML-01-2020-0018

Haobin Ye, B., Fong, L. H. N., & Luo, J. M. (2021). Parasocial interaction on tourism companies' social media sites: antecedents and consequences. *Current Issues in Tourism*, *24*, 1093–1108. https://doi.org/10.1080/13683500.2020.1764915

Harwood, J., Qadar, F., & Chen, C.-Y. (2016). Harmonious contact: Stories about intergroup musical collaboration improve intergroup attitudes. *Journal of Communication*, *66*, 937–959. https://doi.org/10.1111/jcom.12261

Hassim, N., Jayasainan, S. Y., & Khalid, N. L. (2019). Exploring viewer experiences with sageuk K-dramas from a parasocial relations perspective. *SEARCH Journal of Media and Communication Research*, *11*, 77–94.

*+Heins, J., Ruth, N., & Schramm, H. (2017). Gute Jury - Schlechte Jury. Der Einfluss parasozialer Interaktion zwischen Juroren und Zuschauern auf den Erfolg von Musikcastingshows [Good jury - bad jury. The influence of parasocial interaction between jurors and viewers on the success of music casting shows]. In H. Schramm & N. Ruth (Eds.), *Musikcastingshows. Wesen, Nutzung und Wirkung eines populären Fernsehformats [Music casting shows. The nature, use and impact of a popular television format]* (pp. 197–224). Springer.

Hoewe, J., & Sherrill, L. A. (2019). The influence of female lead characters in political TV shows: Links to political engagement. *Journal of Broadcasting & Electronic Media*, *63*, 59–76. https://doi.org/10.1080/08838151.2019.1570782

Hoewe, J., Wiemer, E. C., Adekunle, T., Barton, R., Jett, J., & Pijanowski, A. (2020). Linking political TV shows with female lead characters to political engagement: The roles of parasocial processes and gender identity. *Journal of Broadcasting & Electronic Media*, *64*, 672–692. https://doi.org/10.1080/08838151.2020.1849703

Hoffner, C. A. (2020). Sharing on social network sites following Carrie Fisher's death: Responses to her mental health advocacy. *Health Communication*, *35*, 1475–1486. https://doi.org/10.1080/10410236.2019.1652383

Hoffner, C. A., & Cohen, E. L. (2018). Mental health-related outcomes of Robin Williams' death: The role of parasocial relations and media exposure in stigma, help-seeking, and outreach. *Health Communication*, *33*, 1573–1582. https://doi.org/10.1080/10410236.2017.1384348

Holladay, H. W., & Edgar, A. N. (2019). 'I'm never gonna stop watching it': The paradox of parasocial break-ups in a post-object era. *The Journal of Fandom Studies*, *7*, 213–227. https://doi.org/10.1386/jfs_00001_1

Hornsby, E. R., & Groover, M. (2020). For the love of Jack: Crock-Pot This Is Us and the convergence of crisis communication and parasocial connection. *Florida Communication Journal*, *48*, 21–45.

Hosany, S., Buzova, D., & Sanz-Blas, S. (2020). The influence of place attachment, ad-evoked positive affect, and motivation on intention to visit: Imagination proclivity as a moderator. *Journal of Travel Research*, *59*, 477–495. https://doi.org/10.1177/0047287519830789

Hsu, C.-L. (2020). How vloggers embrace their viewers: Focusing on the roles of para-social interactions and flow experience. *Telematics and Informatics*, *49*, 101364. https://doi.org/10.1016/j.tele.2020.101364

Hu, M. (2016). The Influence of a scandal on parasocial relationship, parasocial interaction, and parasocial breakup. *Psychology of Popular Media Culture*, *5*, 217–231. https://doi.org/10.1037/ppm0000068

Hu, L., Min, Q., Han, S., & Liu, Z. (2020). Understanding followers' stickiness to digital influencers: The effect of psychological responses. *International Journal of Information Management*, *54*, 102169. https://doi.org/10.1016/j.ijinfomgt.2020.102169

Hu, M., Chen, M., Li, M., & Yin, Z. (2019). Meet the media characters from another culture: Influence of ethnocentrism on parasocial interaction. *Psychology of Popular Media Culture*, *8*, 170–179. https://doi.org/10.1037/ppm0000168

Hu, M., Cotton, G., Zhang, B., & Jia, N. (2019). The influence of apology on audiences' reactions toward a media figure's transgression. *Psychology of Popular Media Culture*, *8*, 410–419. https://doi.org/10.1037/ppm0000195

Hu, M., Young, J., Liang, J., & Guo, Y. (2018). An investigation into audiences' reactions to transgressions by liked and disliked media figures. *Psychology of Popular Media Culture*, *7*, 484–498. https://doi.org/10.1037/ppm0000146

Hu, M., Zhang, M., & Wang, Y. (2017). Why do audiences choose to keep watching on live video streaming platforms? An explanation of dual identification framework. *Computers in Human Behavior*, *75*, 594–606. https://doi.org/10.1016/j.chb.2017.06.006

Hu, M., Zhao, Y., Liu, Z., Li, Z., & Kong, X. (2021). Just my imagination: The influence of celebrities' romantic relationship announcements on romance fans and friendship fans. *Psychology of Popular Media*, *10*, 434–444. https://doi.org/10.1037/ppm0000324

Hwang, K., & Zhang, Q. (2018). Influence of parasocial relationship between digital celebrities and their followers on followers' purchase and electronic word-of-mouth intentions, and persuasion knowledge. *Computers in Human Behavior*, *87*, 155–173. https://doi.org/10.1016/j.chb.2018.05.029

Iannone, N. E., McCarty, M. K., Branch, S. E., & Kelly, J. R. (2018). Connecting in the Twitterverse: Using Twitter to satisfy unmet belonging needs. *The Journal of Social Psychology*, *158*, 491–495. https://doi.org/10.1080/00224545.2017.1385445

Ingram, J., & Luckett, Z. (2019). My friend Harry's a wizard: Predicting parasocial interaction with characters from fiction. *Psychology of Popular Media Culture*, *8*, 148–158. https://doi.org/10.1037/ppm0000169

Jahng, M. R. (2019). Watching the rich and famous: the cultivation effect of reality television shows and the mediating role of parasocial experiences. *Media Practice and Education*, *20*, 319–333. https://doi.org/10.1080/25741136.2018.1556544

Jain, P., Pandey, U. S., & Roy, E. (2017). Perceived efficacy and intentions regarding seeking mental healthcare: Impact of Deepika Padukone, a Bollywood celebrity's public announcement of struggle with depression. *Journal of Health Communication*, *22*, 713–720. https://doi.org/10.1080/10810730.2017.1343878

Jain, P., Weed, A., & Walck, P. (2016). From one medium to another: Continuing the narrative world on Twitter and Facebook. *Online Journal of Communication and Media Technologies*, *6*, 74–92.

Jans, S. de, Cauberghe, V., & Hudders, L. (2018). How an advertising disclosure alerts young adolescents to sponsored vlogs: The moderating role of a peer-based advertising literacy intervention through an informational vlog. *Journal of Advertising*, *47*, 309–325. https://doi.org/10.1080/00913367.2018.1539363

Jennings, N., & Alper, M. (2016). Young children's positive and negative parasocial relationships with media characters. *Communication Research Reports*, *33*, 96–102. https://doi.org/10.1080/08824096.2016.1154833

Jenol, N. A. M., & Pazil, N. H. A. (2020). Escapism and motivation: Understanding K-pop fans well-being and identity. *Geografia: Malaysian Journal of Society and Space*, *16*, 336–347. https://doi.org/10.17576/geo-2020-1604-25

Jin, S. V. (2018a). "Celebrity 2.0 and beyond!" Effects of Facebook profile sources on social networking advertising. *Computers in Human Behavior*, *79*, 154–168. https://doi.org/10.1016/j.chb.2017.10.033

Jin, S. V. (2018b). Interactive effects of Instagram foodies' hashtagged #foodporn and peer users' eating disorder on eating intention, envy, parasocial interaction, and online friendship. *Cyberpsychology, Behavior, and Social Networking*, *21*, 157–167. https://doi.org/10.1089/cyber.2017.0476

Jin, S. V., & Ryu, E. (2020). "I'll buy what she's #wearing": The roles of envy toward and parasocial interaction with influencers in Instagram celebrity-based brand endorsement and social commerce. *Journal of Retailing and Consumer Services*, *55*, 102121. https://doi.org/10.1016/j.jretconser.2020.102121

Johnson, E., Rothermilch, K., & Shoenberger, H. (2020). I'll have what she's having: Parasocial communication via social media influences on risk behavior. *The Journal of Social Media in Society*, *9*, 319–334.

Kasap, E. Z., Ağzıtemiz, F., Kızıl, N., & Yıldırım, A. (2018). Understanding the nature of the relationship established with Turkish TV serial characters. *Research Studies Anatolia Journal*, *1*, 237–247.

Kelly, S., Goke, R., McCall, M., & Dowell, S. G. (2020). Parasocial relationships with president Trump as a predictor of COVID-19 information seeking. *Frontiers in Communication*, *5*. https://doi.org/10.3389/fcomm.2020.596663

Kim, C., & Harwood, J. (2019). What makes people imagine themselves in contact with outgroup members: Exploring the relationship between vicarious media contact experiences and imagined contact. *Communication Studies*, *70*, 545–563. https://doi.org/10.1080/10510974.2019.1658612

Kim, C., & Harwood, J. (2020). Parasocial contact's effects on relations between minority groups in a multracial context. *International Journal of Communication*, *14*, 364–385.

Kim, H. (2020). Unpacking unboxing video-viewing motivations: The uses and gratifications perspective and the mediating role of parasocial interaction on purchase intent. *Journal of Interactive Advertising*, *20*, 196–208. https://doi.org/10.1080/15252019.2020.1828202

Kim, J. [Jihyun], Kim, J. [Jinyoung], & Yang, H. (2019). Loneliness and the use of social media to follow celebrities: A moderating role of social presence. *The Social Science Journal*, *56*, 21–29. https://doi.org/10.1016/j.soscij.2018.12.007

Kim, J. [Jihyun], & Song, H. (2016). Celebrity's self-disclosure on Twitter and parasocial relationships: A mediating role of social presence. *Computers in Human Behavior*, *62*, 570–577. https://doi.org/10.1016/j.chb.2016.03.083

Kim, J. [Juran], Kang, S., & Lee, K. H. (2020). How social capital impacts the purchase intention of sustainable fashion products. *Journal of Business Research*, *117*, 596–603. https://doi.org/10.1016/j.jbusres.2018.10.010

Kim, M., & Kim, J. [Jihye]. (2020). How does a celebrity make fans happy? Interaction between celebrities and fans in the social media context. *Computers in Human Behavior*, *111*, 106419. https://doi.org/10.1016/j.chb.2020.106419

Kim, S., Zhang, X. A., & Zhang, B. W. (2016). Self-mocking crisis strategy on social media: Focusing on Alibaba chairman Jack Ma in China. *Public Relations Review*, *42*, 903–912. https://doi.org/10.1016/j.pubrev.2016.10.004

Kishiya, K. (2018). Exploring the impact of celebrity endorsement on product placement effectiveness. *International Journal of Marketing & Distribution*, *2*, 25–35. https://doi.org/10.5844/ijmd.2.1_25

Kosenko, K. A., Binder, A. R., & Hurley, R. (2016). Celebrity influence and identification: A test of the Angelina effect. *Journal of Health Communication*, *21*, 318–326. https://doi.org/10.1080/10810730.2015.1064498

Krause, A. E., North, A. C., & Heritage, B. (2018). Musician interaction via social networking sites: Celebrity attitudes, attachment, and their correlates. *Music & Science*, *1*, 1-11. https://doi.org/10.1177/2059204318762923

Kresovich, A. (2022). The Influence of pop songs referencing anxiety, depression, and suicidal ideation on college students' mental health empathy, stigma, and behavioral intentions. *Health Communication*, *37*, 617–627. https://doi.org/10.1080/10410236.2020.1859724

Kretz, V. E. (2020). McDreamy is McDead: Fan responses to a parasocial break-up. *The Journal of Fandom Studies*, *8*, 147–163. https://doi.org/10.1386/jfs_00014_1

Kurtin, K. S., O'Brien, N., Roy, D., & Dam, L. (2018). The development parasocial interaction relationships on YouTube. *The Journal of Social Media in Society*, *7*, 233–252.

Kurtin, K. S., O'Brien, N., Roy, D., & Dam, L. (2019). Parasocial relationships with musicians. *The Journal of Social Media in Society*, *8*, 30–50.

Kyewski, E., Szczuka, J. M., & Krämer, N. C. (2018). The protagonist, my Facebook friend: How cross-media extensions are changing the concept of parasocial interaction. *Psychology of Popular Media Culture*, *7*, 2–17. https://doi.org/10.1037/ppm0000109

Landreville, K. D., & Niles, C. (2019). "And that's a fact!": The roles of political ideology, PSRs, and perceived source credibility in estimating factual content in partisan news. *Journal of Broadcasting & Electronic Media*, *63*, 177–194. https://doi.org/10.1080/08838151.2019.1622339

Ledbetter, A. M., & Meisner, C. (2021). Extending the personal branding affordances typology to parasocial interaction with public figures on social media: Social presence and media multiplexity as mediators. *Computers in Human Behavior*, *115*, 106610. https://doi.org/10.1016/j.chb.2020.106610

Ledbetter, A. M., & Redd, S. M. (2016). Celebrity credibility on social media: A conditional process analysis of online self-disclosure attitude as a moderator of posting frequency and parasocial interaction. *Western Journal of Communication*, *80*, 601–618. https://doi.org/10.1080/10570314.2016.1187286

Lee, J. E., & Watkins, B. (2016). YouTube vloggers' influence on consumer luxury brand perceptions and intentions. *Journal of Business Research*, *69*, 5753–5760. https://doi.org/10.1016/j.jbusres.2016.04.171

Lee, M.-S., & Park, J. (2017). Television shopping at home to alleviate loneliness among older consumers. *Asia Marketing Journal*, *18*, 139–160. https://doi.org/10.15830/amj.2017.18.4.139

Lee, S. H., Simkins, T. J., Luster, S., & Chowdhury, S. A. (2018). Forgiving sports celebrities with ethical transgressions: The role of parasocial relationships, ethical intent and regulatory focus mindset. *Journal of Global Sport Management*, *3*, 124–145. https://doi.org/10.1080/24704067.2018.1441737

Lee, Y.-H., Yuan, C. W., & Wohn, D. Y. (2021). How video streamers' mental health disclosures affect viewers' risk perceptions. *Health Communication*, *36*, 1931–1941. https://doi.org/10.1080/10410236.2020.1808405

Leksmono, D. L. D. (2016). The process of audience involvement with the media personae on the film "Decendants of the sun" among young female Indonesian viewers. *Journal of Education and Social Sciences*, *4*, 219–224.

Liebers, N., & Schramm, H. (2017). Friends in books: The influence of character attributes and the reading experience on parasocial relationships and romances. *Poetics*, *65*, 12–23. https://doi.org/10.1016/j.poetic.2017.10.001

Liebers, N., & Straub, R. (2020). Fantastic relationships and where to find them: Fantasy and its impact on romantic parasocial phenomena with media characters. *Poetics*, *83*, 101481. https://doi.org/10.1016/j.poetic.2020.101481

Lim, J. S., Choe, M.-J., Zhang, J., & Noh, G.-Y. (2020). The role of wishful identification, emotional engagement, and parasocial relationships in repeated viewing of live-streaming games: A social cognitive theory perspective. *Computers in Human Behavior*, *108*, 106327. https://doi.org/10.1016/j.chb.2020.106327

Lim, M. S., & Kim, J. [Junghyun]. (2018). Facebook users' loneliness based on different types of interpersonal relationships. *Information Technology & People*, *31*, 646–665. https://doi.org/10.1108/ITP-04-2016-0095

Lin, R., Levordashka, A., & Utz, S. (2016). Ambient intimacy on Twitter. *Cyberpsychology: Journal of Psychosocial Research on Cyberspace*, *10*. https://doi.org/10.5817/CP2016-1-6

Lissitsa, S., & Kushnirovich, N. (2020). Is negative the new positive? Secondary transfer effect of exposure to LGBT portrayals in TV entertainment programs. *Journal of Applied Social Psychology*, *50*, 115–130. https://doi.org/10.1111/jasp.12644

Lissitsa, S., & Kushnirovich, N. (2021). Coevolution between parasocial interaction in digital media and social contact with LGBT people. *Journal of Homosexuality*, *68*, 2509–2532. https://doi.org/10.1080/00918369.2020.1809891

Liu, M. T., Liu, Y., & Zhang, L. L. (2019). Vlog and brand evaluations: The influence of parasocial interaction. *Asia Pacific Journal of Marketing and Logistics*, *31*, 419–436. https://doi.org/10.1108/APJML-01-2018-0021

Lou, C., & Kim, H. K. (2019). Fancying the new rich and famous? Explicating the roles of influencer content, credibility, and parental mediation in adolescents' parasocial relationship, materialism, and purchase intentions. *Frontiers in Psychology*, *10*, 2567. https://doi.org/10.3389/fpsyg.2019.02567

Madison, T. P., Covington, E. N., Wright, K., & Gaspard, T. (2019). Credibility and attributes of parasocial relationships with Alex Jones. *Southwestern Mass Communication Journal*, *34*, 1–18.

Madison, T. P., & Porter, L. V. (2016). Cognitive and imagery attributes of parasocial relationships. *Imagination, Cognition and Personality*, *35*, 359–379. https://doi.org/10.1177/0276236615599340

Madison, T. P., Porter, L. V., & Greule, A. (2016). Parasocial compensation hypothesis: Predictors of using parasocial relationships to compensate for real-life interaction. *Imagination, Cognition and Personality*, *35*, 258–279. https://doi.org/10.1177/0276236615595232

Maiorescu, R. D. (2017). Personal public relations and celebrity scandals: A cross-cultural analysis of Twitter communication in the aftermath of Johnny Depp's accusations of domestic violence. *Journal of Communication Management*, *21*, 254–266. https://doi.org/10.1108/JCOM-02-2017-0006

McDermott, D. T., Brooks, A. S., Rohleder, P., Blair, K., Hoskin, R. A., & McDonagh, L. K. (2018). Ameliorating transnegativity: Assessing the immediate and extended efficacy of a pedagogic prejudice reduction intervention. *Psychology & Sexuality*, *9*, 69–85. https://doi.org/10.1080/19419899.2018.1429487

McDonnell, A., & Wheeler, M. (2019). @realDonaldTrump: Political celebrity, authenticity, and para-social engagement on Twitter. *Celebrity Studies*, *10*, 427–431. https://doi.org/10.1080/19392397.2019.1630157

McGregor, S. C. (2018). Personalization, social media, and voting: Effects of candidate self-personalization on vote intention. *New Media & Society*, *20*, 1139–1160. https://doi.org/10.1177/1461444816686103

Munnukka, J., Maity, D., Reinikainen, H., & Luoma-aho, V. (2019). “Thanks for watching”. The effectiveness of YouTube vlogendorsements. *Computers in Human Behavior*, *93*, 226–234. https://doi.org/10.1016/j.chb.2018.12.014

Myrick, J. G. (2019). An experimental test of the roles of audience involvement and message frame in shaping public reactions to celebrity illness disclosures. *Health Communication*, *34*, 1060–1068. https://doi.org/10.1080/10410236.2018.1461170

Myrick, J. G. (2020). Connections between viewing media about President Trump's dietary habits and fast food consumption intentions: Political differences and implications for public health. *Appetite*, *147*, 104545. https://doi.org/10.1016/j.appet.2019.104545

Myrick, J. G., & Erlichman, S. (2020). How audience involvement and social norms foster vulnerability to celebrity-based dietary misinformation. *Psychology of Popular Media*, *9*, 367–379. https://doi.org/10.1037/ppm0000229

Nanda, A. P., & Banerjee, R. (2020). Binge watching: An exploration of the role of technology. *Psychology & Marketing*, *37*, 1212–1230. https://doi.org/10.1002/mar.21353

Natale, D. W. (2017). Parasocial bereavement: Where do they turn when their soap opera friends depart? *Florida Communication Journal*, *45*, 19–33.

Newman, M. J. (2018). My little princess: Exploring mothers’ experiences of their daughter’s parasocial relationships with Disney princesses. *Visual Inquiry*, *7*, 141–151. https://doi.org/10.1386/vi.7.2.141_1

Nisbett, G., & Schartel Dunn, S. (2021). Reputation matters: Parasocial attachment, narrative engagement, and the 2018 Taylor Swift political endorsement. *Atlantic journal of communication*, *29*, 26–38. https://doi.org/10.1080/15456870.2019.1704758

O'Donovan, R. (2016). 'To boldly go where no psychologist has gone before': Effects of participation in fandom activities on parasocial relationships. *Journal of Applied Psychology and Social Science*, *2*, 41–61.

Oliver, M. B., Bilandzic, H., Cohen, J., Ferchaud, A., Shade, D. D., Bailey, E. J. et al. (2019). A penchant for the immoral: Implications of parasocial interaction, perceived complicity, and identification on liking of anti-heroes. *Human Communication Research*, *45*, 169–201. https://doi.org/10.1093/hcr/hqy019

Oschatz, C., & Klimmt, C. (2016). The effectiveness of narrative communication in road safety education: A moderated mediation model. *Communications*, *41*, 145–165. https://doi.org/10.1515/commun-2016-0003

Osterman, L. L., & Hecmanczuk, T. A. (2020). Parasocial forgiveness: The roles of parasocial closeness and offense perceptions. *Journal of Social and Personal Relationships*, *37*, 800–820. https://doi.org/10.1177/0265407519879511

Pan, P.-L., & Zeng, L. (2018). Parasocial interactions with basketball athletes of color in online mediated sports. *Howard Journal of Communications*, *29*, 196–215. https://doi.org/10.1080/10646175.2017.1354790

Paravati, E., Naidu, E., & Gabriel, S. (2021). From “love actually” to love, actually: The sociometer takes every kind of fuel. *Self and identity*, *20*, 6–24. https://doi.org/10.1080/15298868.2020.1743750

Paravati, E., Naidu, E., Gabriel, S., & Wiedemann, C. (2020). More than just a tweet: The unconscious impact of forming parasocial relationships through social media. *Psychology of Consciousness*, *7*, 388–403. https://doi.org/10.1037/cns0000214

Parrott, S., Billings, A. C., Hakim, S. D., & Gentile, P. (2020). From #endthestigma to #realman: Stigma-challenging social media responses to NBA players’ mental health disclosures. *Communication Reports*, *33*, 148–160. https://doi.org/10.1080/08934215.2020.1811365

Pennington, N., Hall, J. A., & Hutchinson, A. (2016). To tweet or not to tweet: Explaining fan-celebrity interaction on Twitter. *Iowa Journal of Communication*, *48*, 55–75.

Perks, L. G., & Turner, J. S. (2019). Podcasts and productivity: A qualitative uses and gratifications study. *Mass Communication and Society*, *22*, 96–116. https://doi.org/10.1080/15205436.2018.1490434

Phua, J. (2016). The effects of similarity, parasocial identification, and source credibility in obesity public service announcements on diet and exercise self-efficacy. *Journal of Health Psychology*, *21*, 699–708. https://doi.org/10.1177/1359105314536452

Phua, J., Lin, J.-S., & Lim, D. J. (2018). Understanding consumer engagement with celebrity-endorsed E-Cigarette advertising on instagram. *Computers in Human Behavior*, *84*, 93–102. https://doi.org/10.1016/j.chb.2018.02.031

Phua, J., & Tinkham, S. (2016). Authenticity in obesity public service announcements: Influence of spokesperson type, viewer weight, and source credibility on diet, exercise, information seeking, and electronic word-of-mouth intentions. *Journal of Health Communication*, *21*, 337–345. https://doi.org/10.1080/10810730.2015.1080326

Purnamaningsih, P., & Rizkalla, N. (2020). The role of parasocial interaction on consumers' intention to purchase beauty products. *Revista CEA*, *6*, 13–27. https://doi.org/10.22430/24223182.1617

Putri, B. M. S., & Yatim, D. I. (2019). Parasocial interaction among young female devotees of Korean dramas. *ANIMA Indonesian Psychological Journal*, *34*, 188–197. https://doi.org/10.24123/aipj.v34i4.2579

Quan, Y., Choe, J. S., & Im, I. (2020). The economics of para-social interactions during live streaming broadcasts : A study of Wanghongs. *Asia Pacific Journal of Information Systems*, *30*, 143–165. https://doi.org/10.14329/apjis.2020.30.1.143

Quintero Johnson, J. M., & Patnoe-Woodley, P. D. (2016). Exploring the influence of parasocial relationships and experiences on radio listeners' consumer behaviors. *Communication Research Reports*, *33*, 40–46. https://doi.org/10.1080/08824096.2015.1117440

Rasmussen, E. E., & Ewoldsen, D. R. (2016). Treatment via television: The relation between watching Dr. Phil and viewers' intentions to seek mental health treatment. *Journal of Health Communication*, *21*, 611–619. https://doi.org/10.1080/10810730.2015.1114054

Rasmussen, L. (2018). Parasocial interaction in the digital age: An examination of relationship building and the effectiveness of YouTube celebrities. *The Journal of Social Media in Society*, *7*, 280–294.

Reinikainen, H., Munnukka, J., Maity, D., & Luoma-aho, V. (2020). 'You really are a great big sister' – parasocial relationships, credibility, and the moderating role of audience comments in influencer marketing. *Journal of Marketing Management*, *36*, 279–298. https://doi.org/10.1080/0267257X.2019.1708781

Reysen, S., Plante, C. N., Roberts, S. E., & Gerbasi, K. C. (2020). Sex differences in parasocial connection to favorite anime characters: A multifactor approach. *The Phoenix Papers*, *4*, 72–92. https://doi.org/10.17605/OSF.IO/E4C5P

Richards, M. N., & Calvert, S. L. (2016). Parent versus child report of young children's parasocial relationships in the United States. *Journal of Children and Media*, *10*, 462–480. https://doi.org/10.1080/17482798.2016.1157502

Richards, M. N., & Calvert, S. L. (2017). Measuring young U.S. children's parasocial relationships: Toward the creation of a child self-report survey. *Journal of Children and Media*, *11*, 229–240. https://doi.org/10.1080/17482798.2017.1304969

*+Riemann, R., Rimscha, M. B. von, Wellbrock, C.-M., & Buschow, C. (2020). Nur Informieren ist nicht genug! Der Einfluss rezipientenseitiger Mediennutzungsmotive auf die Bezahlabsicht für digitaljournalistische Inhalte [Just informing is not enough! The influence of recipients' media usage motives on the intention to pay for digital journalistic content]. In C.-M. Wellbrock & C. Buschow (Eds.), *Money for nothing and content for free? Paid content, Plattformen und Zahlungsbereitschaft im digitalen Journalismus [Money for nothing and content for free? Paid content, platforms and willingness to pay in digital journalism]* (pp. 91–123). Nomos.

Rihl, A., & Wegener, C. (2019). YouTube celebrities and parasocial interaction: Using feedback channels in mediatized relationships. *Convergence*, *25*, 554–566. https://doi.org/10.1177/1354856517736976

Riles, J. M., & Adams, K. (2021). Me, myself, and my mediated ties: Parasocial experiences as an ego-driven process. *Media Psychology*, *24*, 792–813. https://doi.org/10.1080/15213269.2020.1811124

Rodriguez, N. J. (2017). "The Leaver" and "The Left": Sport fans and parasocial divorce. *International Journal of Interactive Communication Systems and Technologies*, *7*, 31–47. https://doi.org/10.4018/IJICST.2017010103

*Rodriguez, N. J. (2020). "It's a Lot Like Ending Up on the Losing Side of a Relationship": Sports Fans, Grief, and Parasocial Divorce. In M. Sarfraz (Ed.), *Innovative Perspectives on Interactive Communication Systems and Technologies* (pp. 241–260). IGI Global. https://doi.org/10.4018/978-1-7998-3355-0.ch012

Rosaen, S. F., & Dibble, J. L. (2016). Clarifying the role of attachment and social compensation on parasocial relationships with television characters. *Communication Studies*, *67*, 147–162. https://doi.org/10.1080/10510974.2015.1121898

Rosaen, S. F., & Dibble, J. L. (2017). The impact of viewer perceptions of media personae and viewer characteristics on the strength, enjoyment, and satisfaction of parasocial relationships. *Communication Studies*, *68*, 1–21. https://doi.org/10.1080/10510974.2016.1240701

Rosaen, S. F., Dibble, J. L., & Hartmann, T. (2019). Does the experience of parasocial interaction enhance persuasiveness of video public service messages? *Communication Research Reports*, *36*, 201–208. https://doi.org/10.1080/08824096.2019.1598854

Ruslan, N., & Abdul Latif, S. (2016). Malay women readings of Korean drama. *Journal of Education and Social Sciences*, *4*, 343–365.

+Ruth, N., Spangardt, B., & Schramm, H. (2016). Time for Talents? Eine Untersuchung von Erfolgsfaktoren bei Musikcastingshows [Time for Talents? An investigation of success factors in music casting shows]. *Musikpsychologie*, *26*, 166–187. https://doi.org/10.23668/psycharchives.2823

*Ryan, P. (2019). Instagram, micro-celebrity and the world of intimate strangers. In C. Palgrave Macmillan (Ed.), *Male sex work in the digital age* (pp. 95–118). Springer.

Sakib, M. N., Zolfagharian, M., & Yazdanparast, A. (2020). Does parasocial interaction with weight loss vloggers affect compliance? The role of vlogger characteristics, consumer readiness, and health consciousness. *Journal of Retailing and Consumer Services*, *52*, 101733. https://doi.org/10.1016/j.jretconser.2019.01.002

Sanderson, J., Zimmerman, M., Stokowski, S., & Fridley, A. (2020). "You had one job!" A case study of maladaptive parasocial interaction and athlete maltreatment in virtual spaces. *International Journal of Sport Communication*, *13*, 221–238. https://doi.org/10.1123/ijsc.2019-0129

Sanz-Blas, S., Bigné, E., & Buzova, D. (2019). Facebook brand community bonding: The direct and moderating effect of value creation behaviour. *Electronic Commerce Research and Applications*, *35*, 100850. https://doi.org/10.1016/j.elerap.2019.100850

Schartel Dunn, S. G. (2018). Parasocial interaction and narrative involvement as predictors of attitude change. *Western Journal of Communication*, *82*, 117–133. https://doi.org/10.1080/10570314.2017.1339230

Schemer, C., & Meltzer, C. E. (2020). The impact of negative parasocial and vicarious contact with refugees in the media on attitudes toward refugees. *Mass Communication and Society*, *23*, 230–248. https://doi.org/10.1080/15205436.2019.1692037

*+Schlütz, D., Lindner, D., & Scheunert, L. (2016). Parasoziale Beziehungen im Web 2.0 - Blogs und Facebook-Fanpage als Beziehungsstifter und Marketinginstrumente [Parasocial relationships in Web 2.0 - blogs and Facebook fan pages as relationship builders and marketing tools]. In G. Zurstiege & D. Schlütz (Eds.), *Sozialität und Werbung [Sociality and advertising]* (pp. 147–171). Herbert von Halem Verlag.

Schnarre, P., & Adam, A. (2018). Parasocial romances as infidelity: Comparing perceptions of real-life, online, and parasocial extradyadic relationships. *Journal of Indiana Academy of the Social Sciences*, *20*, 1–13.

Setyanto, Y., Winduwati, S., & Utami, L. S. S. (2017). Early adolescent behavior on media toward idol figure (parasocial study on preliminary youth as an effect of new media). *International Journal of Communication and Media Studies*, *7*, 1–14.

Shabahang, R., Bagheri Sheykhangafshe, F., Yousefi Siahkoucheh, A., Mokhtari Chirani, B., Mousavi, S. M., & Akhavan, M. (2020). Role of parasocial interaction with narcotic-addicted celebrities and worshipping them in the prediction of addiction potential. *International Journal of Psychology*, *14*, 163–191.

Shan, Y., Chen, K.-J., & Lin, J.-S. (2020). When social media influencers endorse brands: the effects of self-influencer congruence, parasocial identification, and perceived endorser motive. *International Journal of Advertising*, *39*, 590–610. https://doi.org/10.1080/02650487.2019.1678322

Shariffadeen, T. S. A. T. M. A., & Manaf, A. M. A. (2017). Following Islamic reality show personalities on Twitter: A uses and gratification approach to understanding parasocial interaction and social media use. *Intellectual Discourse*, *25*, 637–659.

Shariffadeen, T. S. A. T. M. A., & Manaf, A. M. A. (2020). Malaysian female users' purchase intentions of celebrity-endorsed products on Instagram: A parasocial interaction perspective. *SEARCH Journal of Media and Communication Research*, *12*, 93–109.

Sheldon, Z., Romanowski, M., & Shafer, D. M. (2021). Parasocial interactions and digital characters: the changing landscape of cinema and viewer/character relationships. *Atlantic journal of communication*, *29*, 15–25. https://doi.org/10.1080/15456870.2019.1702550

Shen, L., Seung, S., Andersen, K. K., & McNeal, D. (2018). The psychological mechanisms of persuasive impact from narrative communication. *Studies in Communication Sciences*, *17*, 165–181. https://doi.org/10.24434/j.scoms.2017.02.003

Sherman-Morris, K., Poe, P. S., Nunley, C., & Morris, J. A. (2020). Perceived risk, protective actions and the parasocial relationship with the local weathercaster: A case study of hurricane Irma. *Southeastern Geographer*, *60*, 23–47. https://doi.org/10.1353/sgo.2020.0003

Shin, D.-H. (2016). Do users experience real sociability through social TV? Analyzing parasocial behavior in relation to social TV. *Journal of Broadcasting & Electronic Media*, *60*, 140–159. https://doi.org/10.1080/08838151.2015.1127247

Shin, M., Song, S. W., Kim, S. J., & Biocca, F. (2019). The effects of 3D sound in a 360-degree live concert video on social presence, parasocial interaction, enjoyment, and intent of financial supportive action. *International Journal of Human-Computer Studies*, *126*, 81–93. https://doi.org/10.1016/j.ijhcs.2019.02.001

Silver, N., & Slater, M. D. (2019). A safe space for self-expansion: Attachment and motivation to engage and interact with the story world. *Journal of Social and Personal Relationships*, *36*, 3492-3514. https://doi.org/10.1177/0265407519826345

Slater, M. D., Ewoldsen, D. R., & Woods, K. W. (2018). Extending conceptualization and measurement of narrative engagement after-the-fact: Parasocial relationship and retrospective imaginative involvement. *Media Psychology*, *21*, 329–351. https://doi.org/10.1080/15213269.2017.1328313

*Smith, K. (2018). More than a comedian: Exploring cybermourning and parasocial relationships in the night Hollywood star Robin Williams died. In Y. Kamalipour (Ed.), *Global discourses in fractured time: Perspectives on journalism, media, education, and politics* (pp. 1–27). Cambridge Scholars Publishing.

Smith-Frigerio, S. (2018). Intersectionality of race, class and gender: The complex representation of bipolar disorder on Fox Network's Empire. *Howard Journal of Communications*, *29*, 387–402. https://doi.org/10.1080/10646175.2017.1407720

So, J., & Shen, L. (2016). Personalization of risk through convergence of self- and character-risk: Narrative effects on social distance and self-character risk perception gap. *Communication Research*, *43*, 1094–1115. https://doi.org/10.1177/0093650215570656

Sokolova, K., & Kefi, H. (2020). Instagram and YouTube bloggers promote it, why should I buy? How credibility and parasocial interaction influence purchase intentions. *Journal of Retailing and Consumer Services*, *53*, 101742. https://doi.org/10.1016/j.jretconser.2019.01.011

Sokolova, K., & Perez, C. (2021). You follow fitness influencers on YouTube. But do you actually exercise? How parasocial relationships, and watching fitness influencers, relate to intentions to exercise. *Journal of Retailing and Consumer Services*, *58*, 102276. https://doi.org/10.1016/j.jretconser.2020.102276

Song, W., & Fox, J. (2016). Playing for love in a romantic video game: Avatar identification, parasocial relationship, and Chinese women's romantic beliefs. *Mass Communication and Society*, *19*, 197–215. https://doi.org/10.1080/15205436.2015.1077972

Sorlin, S. (2018). Strategies of involvement and moral detachment in House of Cards. *Journal of Literary Semantics*, *47*, 21–41. https://doi.org/10.1515/jls-2018-0002

Spangardt, B., Ruth, N., & Schramm, H. (2016). "… and please visit our Facebook page, too!" How radio presenter personalities influence listeners' interactions with radio stations. *Journal of Radio & Audio Media*, *23*, 68–94. https://doi.org/10.1080/19376529.2016.1155710

Stein, J.-P., Koban, K., Joos, S., & Ohler, P. (2020). Worth the effort? Comparing different youtube vlog production styles in terms of viewers' identification, parasocial response, immersion, and enjoyment. *Psychology of Aesthetics, Creativity, and the Arts*. https://doi.org/10.1037/aca0000374

Stever, G. S. (2017). Evolutionary theory and reactions to mass media: Understanding parasocial attachment. *Psychology of Popular Media Culture*, *6*, 95–102. https://doi.org/10.1037/ppm0000116

Sycoff, L., & Cunningham, C. (2020). An idiopathic dalliance in television, para-social relationships, and consumption: A study of pop-culture television nutrition and the bandwagon effect. *Journal of Student Research*, *9*. https://doi.org/10.47611/jsrhs.v9i2.1086

Taber, L., Baltaxe-Admony, L. B., & Weatherwax, K. (2019). What makes a live stream companion? Animation, beats, and parasocial relationships. *Interactions*, *27*, 52–57. https://doi.org/10.1145/3372042

Tal-Or, N., & Razpurker-Apfeld, I. (2021). When the physical coldness in the viewer's environment leads to identification with a suffering protagonist. *International Journal of Psychology*, *56*, 394–406. https://doi.org/10.1002/ijop.12722

Tolbert, A. N., & Drogos, K. L. (2019). Tweens' wishful identification and parasocial relationships with YouTubers. *Frontiers in Psychology*, *10*, 2781. https://doi.org/10.3389/fpsyg.2019.02781

Tran, G. A., Yazdanparast, A., & Strutton, D. (2019). Investigating the marketing impact of consumers' connectedness to celebrity endorsers. *Psychology & Marketing*, *36*, 923–935. https://doi.org/10.1002/mar.21245

Tsai, W.-H. S., & Men, L. R. (2017). Social CEOs: The effects of CEOs' communication styles and parasocial interaction on social networking sites. *New Media & Society*, *19*, 1848–1867. https://doi.org/10.1177/1461444816643922

Tsiotsou, R. H. (2016). The social aspects of consumption as predictors of consumer loyalty. *Journal of Service Management*, *27*, 91–116. https://doi.org/10.1108/JOSM-04-2015-0117

Tukachinsky, R. (2020). Playing a bad character but endorsing a good cause: Actor-character fundamental attribution error and persuasion. *Communication Reports*, *33*, 1–13. https://doi.org/10.1080/08934215.2019.1691618

Tukachinsky, R., Brogan-Freitas, E., & Urbanovich, T. (2019). Promoting support for public health policies through mediated contact: Can narrator perspective and self-disclosure curb in-group favoritism. *International Journal of Communication*, *13*, 4553–4571.

Tukachinsky, R., & Dorros, S. M. (2018). Parasocial romantic relationships, romantic beliefs, and relationship outcomes in USA adolescents: rehearsing love or setting oneself up to fail? *Journal of Children and Media*, *12*, 329–345. https://doi.org/10.1080/17482798.2018.1463917

Tukachinsky, R., & Eyal, K. (2018). The psychology of marathon television viewing: Antecedents and viewer involvement. *Mass Communication and Society*, *21*, 275–295. https://doi.org/10.1080/15205436.2017.1422765

Tukachinsky, R., & Sangalang, A. (2016). The effect of relational and interactive aspects of parasocial experiences on attitudes and message resistence. *Communication Reports*, *29*, 175–188. https://doi.org/10.1080/08934215.2016.1148750

Vazquez, D., Cheung, J., & Wu, X. (2019). Investigating Chinese audience-consumer responses towards TC character-based fashion related social media content. *International Journal of Business and Globalisation*, *22*, 53–73.

Vazquez, D., Wu, X., Nguyen, B., Kent, A., Gutierrez, A., & Chen, T. (2020). Investigating narrative involvement, parasocial interactions, and impulse buying behaviours within a second screen social commerce context. *International Journal of Information Management*, *53*, 102135. https://doi.org/10.1016/j.ijinfomgt.2020.102135

Vickery, A. J., & Ventrano, S. (2021). Listening goals and parasocial relationships: How listening styles impact the development of parasocial relationships with media personas. *International Journal of Listening*, *35*, 149–165. https://doi.org/10.1080/10904018.2020.1781637

Vinney, C., & Vinney, L. A. (2017). That sounds familiar: The relationship between listeners' recognition of celebrity voices, perceptions of vocal pleasantness, and engagement with media. *Journal of Radio & Audio Media*, *24*, 320–338. https://doi.org/10.1080/19376529.2017.1346659

Visintin, E. P., Voci, A., Pagotto, L., & Hewstone, M. (2017). Direct, extended, and mass-mediated contact with immigrants in Italy: their associations with emotions, prejudice, and humanity perceptions. *Journal of Applied Social Psychology*, *47*, 175–194. https://doi.org/10.1111/jasp.12423

Vonderohe, B. M. (2016). Twitter's role in the disclosure of parasocial relationships. *UW-L Journal of Undergraduate Research*.

Waggoner, E. B. (2022). Impact of disclosure videos and self-understanding imagined interactions on emotions and homophobia. *Journal of Homosexuality*, *69*, 169–189. https://doi.org/10.1080/00918369.2020.1815427

Wahab, H. K. A., & Tao, M. (2019). The influence of internet celebrity on purchase decision and materialism: The mediating role of para-social relationships and identification. *European Journal of Business and Management*, *11*, 183–199. https://doi.org/10.7176/EJBM/11-15-20

Ward, J. (2016). A content analysis of celebrity Instagram posts and parasocial interaction. *Elon Journal of Undergraduate Research in Communications*, *7*.

Wasike, B. (2018). Gender, parasocial interaction, and nonverbal communication: Testing the visual effect of sports magazine cover models. *International Journal of Communication*, *12*.

Wellman, M. L. (2021). Trans-mediated parasocial relationships: Private Facebook groups foster influencer–follower connection. *New Media & Society*, *23*, 3557–3573. https://doi.org/10.1177/1461444820958719

Wen, N. (2017). Celebrity influence and young people's attitudes toward cosmetic surgery in Singapore: The role of parasocial relationships and identification. *International Journal of Communication*, *11*, 1234–1252.

+Wiethe, L., Rudeloff, C., & Tellkampf, U. (2020). Zwischen Unterhaltung und Interaktion: Über das Warum der Podcast-Nutzung [Between entertainment and interaction: On the why of podcast use]. *kommunikation@gesellschaft, 21*. https://doi.org/10.15460/kommges.2020.21.2.629

Wong, D. T. L., & Patlamazoglou, L. (2022). Bereavement and coping following the death of a personally significant popular musician. *Death Studies*, *46*, 1234–1242. https://doi.org/10.1080/07481187.2020.1809031

Wong, N. C. H., Lookadoo, K. L., & Nisbett, G. S. (2017). "I'm Demi and I have bipolar disorder": Effect of parasocial contact on reducing stigma toward people with bipolar disorder. *Communication Studies*, *68*, 314–333. https://doi.org/10.1080/10510974.2017.1331928

Woznicki, N., Arriaga, A. S., Caporale-Berkowitz, N. A., & Parent, M. C. (2021). Parasocial relationships and depression among LGBQ emerging adults living with their parents during COVID-19: The potential for online support. *Psychology of Sexual Orientation and Gender Diversity*, *8*, 228–237. https://doi.org/10.1037/sgd0000458

Wu, S. (2020). An empirical research on social media marketing and consumer responses: Leveraging the power of online opinion leaders. *The Kyoto Economic Review*, *87*, 34–63. https://doi.org/10.11179/ker.87.34

Wu, Y., Mou, Y., Wang, Y., & Atkin, D. (2018). Exploring the de-stigmatizing effect of social media on homosexuality in China: An interpersonal-mediated contact versus parasocial-mediated contact perspective. *Asian Journal of Communication*, *28*, 20–37. https://doi.org/10.1080/01292986.2017.1324500

Wulf, T., & Rieger, D. (2018). Wallowing in media past: Media-induced nostalgia's connection to parasocial relationships. *Communication Research Reports*, *35*, 178–182. https://doi.org/10.1080/08824096.2017.1383236

Wulf, T., Schneider, F. M., & Beckert, S. (2020). Watching players: An exploration of media enjoyment on Twitch. *Games and Culture*, *15*, 328–346. https://doi.org/10.1177/1555412018788161

Xiang, L., Zheng, X., Lee, M. K., & Zhao, D. (2016). Exploring consumers' impulse buying behavior on social commerce platform: The role of parasocial interaction. *International Journal of Information Management*, *36*, 333–347. https://doi.org/10.1016/j.ijinfomgt.2015.11.002

Xu, X., Wu, J. H., & Li, Q. (2020). What drives consumer shopping behavior in live streaming commerce? *Journal of Electronic Commerce Research*, *21*, 144–167.

Yan, Q., & Yang, F. (2021). From parasocial to parakin: Co-creating idols on social media. *New Media & Society*, *23*, 2593–2615. https://doi.org/10.1177/1461444820933313

*Yang, Y. (2019). Self-presentation, interaction, and marketing of Chinese athletes on social media: A study of men's national table tennis team. In M. A. Dos Santos (Ed.), *Integrated Marketing Communications, Strategies, and Tactical Operations in Sports Organizations* (pp. 48–67). IGI Global.

Yuan, C. L., Kim, J. [Juran], & Kim, S. J. (2016). Parasocial relationship effects on customer equity in the social media context. *Journal of Business Research*, *69*, 3795–3803. https://doi.org/10.1016/j.jbusres.2015.12.071

Yuan, C. L., Moon, H., Kim, K. H., & Wang, S. (2021). The influence of parasocial relationship in fashion web on customer equity. *Journal of Business Research*, *130*, 610–617. https://doi.org/10.1016/j.jbusres.2019.08.039

Yuan, S., & Lou, C. (2020). How social media influencers foster relationships with followers: The roles of source credibility and fairness in parasocial relationship and product interest. *Journal of Interactive Advertising*, *20*, 133–147. https://doi.org/10.1080/15252019.2020.1769514

Yuksel, M., & Labrecque, L. I. (2016). "Digital buddies": Parasocial interactions in social media. *Journal of Research in Interactive Marketing*, *10*, 305–320. https://doi.org/10.1108/JRIM-03-2016-0023

Zafar, A. U., Qiu, J., & Shahzad, M. (2020). Do digital celebrities' relationships and social climate matter? Impulse buying in f-commerce. *Internet Research*, *30*, 1731–1762. https://doi.org/10.1108/INTR-04-2019-0142

Zafar, Z., Ali, F., Awais, M., & Saeed, M. (2020). Exposure to mediated celebrities and mate preferences: The mediating role of identification and parasocial relationships. *Journal of Organizational Culture, Communications and Conflicts*, *24*, 1–16.

Zaheer, L. (2019). Parasocial interaction and television viewers in Pakistan. *Journal of Behavioural Sciences*, *29*, 38–52.

Zhang, H., Xu, H., & Gursoy, D. (2020). The effect of celebrity endorsement on destination brand love: A comparison of previous visitors and potential tourists. *Journal of Destination Marketing & Management*, *17*, 100454. https://doi.org/10.1016/j.jdmm.2020.100454

Zhang, K. (2020). The effect of para-social interaction in endorsement advertising: SEM studies based on consumers' exposure to celebrity symbols. *Language and Semiotic Studies*, *6*, 42–77.

Zhang, K., & Hung, K. (2020). The effect of natural celebrity–brand association and para-social interaction in advertising endorsement for sustainable marketing. *Sustainability*, *12*, 6215. https://doi.org/10.3390/su12156215

Zsila, Á., Urbán, R., McCutcheon, L. E., & Demetrovics, Z. (2021). A new avenue to reach out for the stars: The association of celebrity worship with problematic and nonproblematic social media use. *Psychology of Popular Media*, *10*, 105–114. https://doi.org/10.1037/ppm0000275
