# Supplementary material for: Research trends on parasocial interactions and relationships with media characters. A review of 281 English and German-language studies from 2016 to 2020
Source: Front Psychol. 2024 Sep 26;15:1418564. doi: 10.3389/fpsyg.2024.1418564 (PMC11464444; doi:10.3389/fpsyg.2024.1418564)
Supplement: Supplementary file 2 [file Table_2.pdf]

Appendix 2: Coding Scheme

Language
1 = English
2 = German

Type of publication
1 = Journal article
2 = Book chapter
3 = Monograph

Year of publication

Media category
1 = Film and television
2 = Print media
3 = Radio, music and podcasts
4 = Social and new media
5 = Cross-media

Fictionality
1 = fictional
2 = non-fictional
3 = fictional and non-fictional

Construct
Each construct as a separate column and then code dichotomously for each study whether present or not (0 = not present; 1 = present):
- PSI
- PSR
- PSRB
- Friendly
- Romantic
- Negative

Positioning in the hypothesis model
1 = Cause
2 = Mediator/Moderator
3 = Effect
4 = Neither

Qualitative versus quantitative research
1 = Qualitative
2 = Quantitative
3 = both

Method
1 = Survey
2 = Experiment
3 = Content analysis
4 = Observation

5 = Narrative analysis
6 = In-depth interviews
7 = Several different data collection methods

Measurement
1 = Development of an own scale
2 = Use of an existing measurement or an adapted version of it
3 = Use of different measurement instruments
4 = Use of an existing measurement and own items
5 = No quantitative measurement of the construct
6 = Development of own scale and use of an existing measurement
7 = Own category system
8 = Self-formulated items
9 = No measurement, neither qualitative nor quantitative
10 = No specification

Age of the majority of the sample
1 = Infants (0-4 years)
2 = Children (5-12 years)
3 = Adolescents (13-17 years)
4 = Budding adults (18-24 years)
5 = Young adults (25-34 years)
6 = Full adults (35-59 years)
7 = Older adults (60 years and older)
8 = Adults not specified (18 years and older without specification)
9 = Various age groups
10 = No data collection with persons
11 = Several data collections with and without persons

Gender of the majority of the sample
1 = Balanced (45-55% female)
2 = Rather male (25-44% female)
3 = Rather female (56-75% female)
4 = Predominantly male (1-24% female)
5 = Predominantly female (76-99% female)
6 = Exclusively male (0% female)
7 = Exclusively female (100% female)
8 = Mixed without further specification
9 = Various sample compositions
10 = No data collection with persons
11 = Several data collections with and without persons
